# A case of mixed histiocytosis (Erdheim-Chester disease and Langerhans cell histiocytosis) with STEAP3-associated anemia and type 4 hemochromatosis

**DOI:** 10.1186/s13023-026-04379-2

**Published:** 2026-05-11

**Authors:** Anastasiia A. Buianova, Tatiana A. Gaydina, Elena V. Reznik, Maksim D. Iarovoi, Anna A. Kuznetsova, Vera A. Belova, Mikhail A. Ignatyuk, Petr A. Shatalov, Zhanna A. Repinskaia, Anna P. Shinkarina, Artem V. Volodkin, Dmitrii A. Atiakshin, Dmitriy O. Korostin

**Affiliations:** 1 Genomics Laboratory, Pirogov Russian National Research Medical University, 1, bld. 1, Ostrovityanova St, Moscow, 117513 Russia; 2 Department of Dermatovenereology named after Academician Yu.K. Skripkin, Institute of Clinical Medicine, Pirogov Russian National Research Medical University, 1, bld. 1, Ostrovityanova St, Moscow, 117513 Russia; 3 Department of Propaedeutics of Internal Medicine, Institute of Clinical Medicine, Pirogov Russian National Research Medical University, No. 2, 1, bld. 1, Ostrovityanova St, Moscow, 117513 Russia; 4 RUDN University, 6 Miklukho-Maklaya St, Moscow, 117198 Russia; 5 National Medical Research Radiological Centre of the Ministry of Health of the Russian Federation, 4 Koroleva St, Obninsk, 249036 Russia; 6 Center for Precision Genome Editing and Genetic Technologies for Biomedicine, Pirogov Russian National Research Medical University, 1, bld. 1, Ostrovityanova St, Moscow, 117513 Russia

**Keywords:** Mixed histiocytosis, BRAF, Langerhans cells, Erdheim-Chester disease, Anemia, Hemochromatosis

## Abstract

**Background:**

Mixed histiocytosis (MH), the coexistence of two or more histiocytic disorders in the same patient, is rare and poorly understood. Langerhans cell histiocytosis (LCH) is a clonal myeloid disorder characterized by infiltration of pathological antigen-presenting cells with morphological and phenotypic resemblance to Langerhans cells. Activating mutations of the MAPK/ERK signaling cascade represent the principal driver of LCH pathogenesis and are also central to Erdheim-Chester disease (ECD).

**Methods:**

We present a 34-year-old patient diagnosed with a mixed form of LCH and ECD involving the kidneys, skin, bones, ENT organs, eyes, and central nervous system. The clinical phenotype included diabetes insipidus, chronic otitis externa, and suspected hereditary anemia and hemochromatosis. Tissue samples were examined using histochemical, immunohistochemical, and immunofluorescence methods, along with whole-exome sequencing (WES) of blood and skin. The patient demonstrated a partial clinical and laboratory response to targeted therapy with trametinib and desmopressin.

**Results:**

Comparison of WES data from blood and skin revealed 71,953 shared variants consistent with likely germline origin, 3,081 somatic variants in skin, and 2,633 in blood. A low-level mosaic *BRAF* V600E mutation (variant allele frequency 1%) was identified in skin, in addition to pathogenic germline variants in *STEAP3* (c.523–2 A > T, NM_182915.3) and *SLC40A1* (c.332T > A, p.Met111Lys, NM_014585.6). Immunofluorescence of bone marrow trephine biopsy demonstrated heterogeneous cellular populations: sparse tryptase-positive mast cells with minimal intercellular contacts; Vimentin^+^CD11b^–^CD34^+^ (0.68%) and Vimentin^+^CD11b^+^CD34^–^ (7.78%) stromal subsets; and a predominance of CD68^+^ cells (55.61%) over S100^+^ cells (43.02%). Double-positive CD68^+^S100^+^ cells were infrequent (2.174%), consistent with transitional stages of Langerhans cells toward LCH cells (up to 4.81%). This hybrid microenvironment paralleled the patient’s systemic inflammatory activity (elevated acute-phase reactants), multi-organ involvement, and renal interstitial fibrosis/tubular atrophy with impaired kidney function, without serological or histological evidence of vasculitis.

**Conclusion:**

This case illustrates the histological heterogeneity and hybrid nature of MH, where canonical dendritic LCH cells coexist with macrophage-rich ECD-like infiltrate. An integrated approach involving a multidisciplinary clinical team, thorough molecular genetic testing, and the application of targeted therapy is essential for improving prognosis and quality of life in young patients.

**Supplementary Information:**

The online version contains supplementary material available at 10.1186/s13023-026-04379-2.

## Introduction

Increasing attention has been given to mixed histiocytosis (MH), a condition in which two or more histiocytic disorders occur in the same patient either synchronously or sequentially. In a recent multicenter cohort study, Pegoraro et al. reported that 14% of patients with Erdheim-Chester disease (ECD) (69/502) had mixed ECD-Langerhans cell histiocytosis (LCH), which more frequently presents as a multisystem disease involving long bones, central nervous system, lungs, skin, orbitofacial structures, and the hypothalamic–pituitary axis, and is often associated with the *BRAF* V600E mutation [1]. Similarly, Dai et al. found that patients with mixed LCH/ECD tend to be younger at diagnosis and exhibit disseminated disease with multiple organs affected, including bones, lungs, central nervous system (CNS), retroperitoneum, and large vessels [2].

Systematic analyses have shown that more than 100 cases of MH have been described in the literature, with the most frequent combination being LCH and ECD [3]. Based on clinical presentation and temporal relationships between lesions, three principal forms of MH have been proposed: Type-1 (sensu stricto), characterized by synchronous or metachronous coexistence of LCH and non-Langerhans histiocytosis – most commonly ECD – usually presenting as a multisystem disease; Type-2, a therapy-related form in which non-Langerhans histiocytic lesions such as juvenile or adult xanthogranuloma develop after systemic treatment of LCH; and Type-3, typically a single-system disease in which both LCH and non-Langerhans histiocytic cells are identified within the same lesion at histopathological examination [3].

LCH belongs to the group of histiocytic neoplasms and is characterized by clonal proliferation of antigen-presenting cells exhibiting the Langerhans cell (LC) phenotype. Lesions typically consist of pathological LCs with Birbeck granules, T-lymphocytes, eosinophils, and macrophages. Pathological LCs express CD1a^++^ and CD207^+++^ similarly to normal epidermal LCs but are less differentiated [4]. Immunohistochemical (IHC) analysis of lesional tissue reveals expression of CD1a (membranous), S100 (nuclear and cytoplasmic), CD207/langerin (granular cytoplasmic) and CD68 (Golgi dot-like). The Ki-67 proliferation index usually ranges below 10% [5]. Absence of expression for CD1c, B- and T-cell markers, CD23, and CD30 is expected.

Macrophages and dendritic cells (including LC) may originate from CD34^+^ hematopoietic stem cells in bone marrow (including transplanted marrow) or circulating monocytes. Initial histiocytosis classification relied on Langerhans (CD1a, CD207) vs. macrophage (CD68, CD163) markers, but hybrid forms have since been described (e.g., combined LCH, ECD, and Rosai-Dorfman-Destombes disease [RDD]) often sharing a single mutation, suggesting common origin [6]. Additionally, single LCH lesions can contain heterogeneous cell populations [7]. It is hypothesized that LCs derive from oligopotent bone marrow progenitors, recruited to inflamed tissues, where they differentiate into pathological cells [8]. A recent study suggests DC3/monocytes as LCH precursors, based on iPSC models with *BRAF* V600E mutation that differentiated into CD14^low^/CD1c^+^ precursors, then into CD1a^+^/CD207^+^ LCH-like cells [9].

LCH incidence in children under 15 ranges from 2.6 to 8.9 per million per year (median diagnosis age: 3 years [10]; M:F ratio = 1.2:1 [4]), while incidence in adults is estimated at 1–2 cases per million annually [11]. Pediatric LCH is often systemic with fever, hepatosplenomegaly, cytopenia, and sometimes deafness or chronic otorrhea; adults show more pulmonary involvement (dyspnea, cough), tooth loss, while diabetes insipidus occurs in both groups [6]. Adolescents (14–17 years) present with adult-like features: more frequent pituitary and pulmonary involvement, less cutaneous and hematologic manifestations [12].

Although hereditary etiology is unconfirmed, a genome-wide association study of familial LCH identified variant rs12438941 in *SMAD6* as significantly associated with disease risk. SMAD6 inhibits BMP/TGF-β pathways involved in LC differentiation; thus, polymorphisms may dysregulate myeloid differentiation [13].

Modern molecular research has shown that activating mutations in the MAPK (mitogen-activated protein kinase)/ERK (extracellular signal-regulated kinase) pathway are central to LCH pathogenesis. The most frequent and clinically relevant mutation is *BRAF* V600E (exon 15), which constitutively activates the BRAF kinase domain independently of RAS signaling [14, 15]. This mutation is found in ~ 50–65% of LCH cases and is classified as oncogenic (Level 2; FDA Level 3), responsive to selective BRAF inhibitors such as vemurafenib and dabrafenib [16]. Notably, *BRAF* V600E is detectable in trephine biopsies and myeloid precursors from blood but absent in peripheral mononuclear cells, supporting its somatic nature [4]. *BRAF* V600E in early myeloid cells (CD11c^+^, CD14^+^, CD34^+^) correlates with multisystem involvement (liver, spleen, bone marrow) and higher relapse risk, while its restriction to differentiated cells (CD207^+^ only) is associated with localized disease and better prognosis [17]. MEK inhibitors such as cobimetinib and trametinib are effective in LCH with MAPK-pathway mutations (*BRAF*, *MAP2K1*, *NRAS*) and recommended as monotherapy by NCCN. *MAP2K1* mutations (10–20%) activate ERK1/2 and are second most frequent, while *NRAS* mutations are rarer [18–20]. In a cohort of 99 Russian LCH patients, 44% were *BRAF*-positive [21]. Genes analyzed included *BRAF*, *ARAF*, *MAP2K1*, *MAP3K1*, *NRAS*, *PIK3CA*, *KIT*, *ERBB3*, *KRAS*, *PIK3CD*, and *HRAS*.

Unlike early phenotype-based classification, the current system [22] is based on clinical-radiological features and organ involvement, enhancing clinical utility. Four LCH subtypes are defined: unifocal (single-organ), unisystem pulmonary (lung-only, often smoking-related), unisystem multifocal (> 1 site in a single organ), and multisystem (> 2 organs/systems).

LCH diagnosis is established via clinical presentation, histopathological evaluation, computed tomography (CT)/magnetic resonance imaging (MRI) (for bone, lung, CNS assessment), and molecular-genetic testing (Table 1) [23].

**Table 1:** Clinical manifestations of LCH [24]

Affected Organ/System	Clinical and Radiologic Features
Bones	Lytic bone lesions observed in 30–50% of cases on positron emission tomography-computed tomography (PET-CT).
Skin	Erythematous, papular eruptions; less commonly, involvement of oral, genital, or perianal mucosa.
Endocrine system	Pituitary insufficiency.
Nervous system	Focal or extensive lesions of the pituitary stalk, hypothalamus, or pineal gland. Less commonly, infiltration of the brainstem or cerebellum with development of ataxia and dysarthria.
Lungs	Cystic or nodular formations presenting with obstructive, restrictive, or mixed changes, pneumothorax.
Liver, spleen	Early-stage: parenchymal infiltration (hepatomegaly, tumor-like nodules, mild cholestasis). Late-stage: sclerosing cholangitis-like damage (severe cholestasis) rapidly progressing to terminal liver failure and death.
Bone marrow	Involvement with changes in complete blood count.
Lymph nodes	Lymphadenopathy.
Gastrointestinal tract	Diarrhea, abdominal discomfort, colonic polyps.
Cardiovascular system	Arterial stenosis, more common in mixed forms.

Treatment depends on disease extent, organ involvement, and molecular profile [23]. Evidence suggests that MAPK inhibitors effectively reduce mature CD14^+^ cell populations but do not affect myeloid progenitors [9]. Patients with Type-3 MH, which typically presents as single-system lesions, may benefit from limited surgical excision, while Type-1 and Type-2 MH frequently harbor *BRAF* V600E mutations and may respond to targeted BRAF or MEK inhibitors, particularly when conventional therapies result only in partial remission or stable disease [3]. Targeted therapy with BRAF and MEK inhibitors has shown high efficacy, with Pegoraro et al. reporting responses in 75% of mixed ECD-LCH patients, and Dai et al. confirming durable responses in first- or second-line therapy for *BRAF* V600E-positive cases [1, 2]. Conventional treatments, including interferon-α and chemotherapy, are less effective in mixed forms, especially in patients with CNS or cardiovascular involvement. Cladribine or cytarabine may be used for risk organ involvement (liver, spleen, bone marrow). Hormone replacement is indicated for pituitary insufficiency, and pulmonary rehabilitation may benefit patients with lung fibrosis. Five-year survival for localized disease exceeds 90%, whereas prognosis in multisystem disease largely depends on therapy response and relapse frequency. Key predictors of outcome include age at diagnosis, presence of hematologic comorbidities, treatment failure, and type of bone involvement, with lytic lesions associated with better survival [1]. Follow-up should include serial imaging (FDG-PET/CT, MRI/CT), endocrine evaluation, and organ-specific monitoring, particularly for patients receiving targeted therapy [25].

## Case report

Patient N., a 34-year-old male, had been under observation by specialists of various disciplines since 2019 due to a progressive array of symptoms.

Anamnesis vitae: Full-term birth from an uncomplicated pregnancy. Normal growth and developmental milestones. Holds a university degree; currently unemployed. From 1986 to 1998, resided in an area classified as a zone with preferential socio-economic status due to radioactive contamination by cesium-137 (1–5 Ci/m^2^) following the Chernobyl disaster. Smoked up to 2 cigarettes per day for 10 years; quit in December 2024. Denies history of tuberculosis, sexually transmitted infections, or viral hepatitis. Vaccinated per national immunization schedule. Denies prior trauma, surgeries, or excessive sun exposure. Medical history includes varicella and upper respiratory infections. No known hereditary or allergic conditions.

Anamnesis morbi: The patient considers himself ill since 2019, when he first experienced headaches and dizziness. He consulted a local general practitioner and was diagnosed with arterial hypertension (maximum blood pressure 160/120 mmHg). Prescribed perindopril 5 mg/day. Simultaneously, he noted severe thirst but did not undergo further evaluation.

By 2020, the patient developed ear pain and significant discharge of white epidermoid material from the external auditory canals. Chronic bilateral otitis media was diagnosed. Topical antibacterial treatment yielded partial symptom relief, reducing pain but not otorrhea. Otolaryngological cleaning of the ear canals was performed every 3–4 months.

In spring 2022, the patient began experiencing persistent, intense headaches (mainly in the right parietal region) with a shooting character. Treatment with B vitamins, nootropics, and nonsteroidal anti-inflammatory drugs (NSAIDs) produced only temporary relief. Around the same time, he reported pain in the right shoulder and elbow joints. Rheumatoid factor was within normal limits. A rheumatologist prescribed chondroprotectors, muscle relaxants, and a 3-month course of therapy with mild improvement.

By summer 2022, the patient noticed ulcerations on the oral mucosa and erosions in the groin area. He used topical antibacterial and antifungal agents with minimal effect.

In December 2022, an endocrinologist diagnosed central diabetes insipidus. No pharmacological treatment was initiated; further investigation was recommended.

**Fig. 1:**
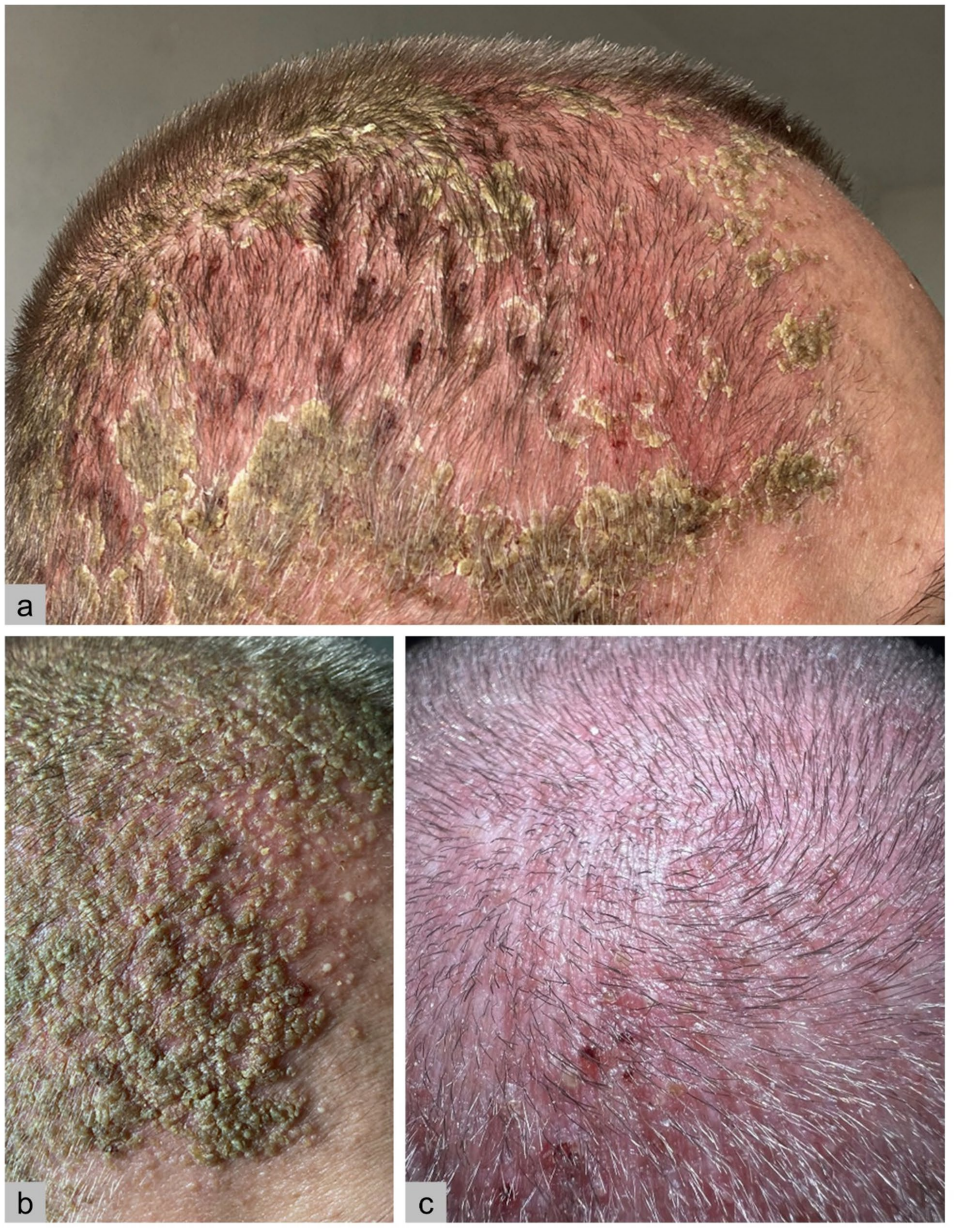
**a**, **b** – Macroscopic images of the patient’s scalp (2023): small papules and pustules on an erythematous background, numerous confluent massive serous crusts. **c** – Macroscopic image of the scalp after initiation of trametinib therapy (2025)

In January 2023, the patient consulted a dermatologist due to a scalp rash with dense crust formation (Fig. 1a, b). Dermatological evaluation: The patient reported itchy rashes on the scalp, groin folds, and lower legs. Status localis: On the scalp, the pathological changes included small papules and pustules on an erythematous background with numerous confluent massive serous crusts. Hair was of normal structure without pathological changes. Bilateral groin folds exhibited maceration and linear erosions. Skin of both lower legs appeared dry, tense, with scaling and excoriations. Differential diagnosis included seborrheic dermatitis, psoriasis, and dermatomycosis. Topical treatments with antibacterial, antifungal, corticosteroid, and combination therapies (ketoconazole, betamethasone dipropionate with salicylic acid, sertaconazole, dioxomethyltetrahydropyrimidine with chloramphenicol, clotrimazole + beclomethasone dipropionate + gentamicin sulfate, methylprednisolone aceponate) showed no effect. Minor improvement in the groin area was noted following treatment with 1% aqueous methylene blue solution.

In February 2023, the patient experienced a recurrence of rashes and erosions in the inguinal area and on the oral mucosa. Intramuscular injections of calcium gluconate were prescribed with minimal clinical effect.

In spring 2023, there was a recurrence of arthralgia in the elbow and shoulder joints, as well as headaches.

In April 2024, the patient reported episodes of white flashes of light in both eyes. Ophthalmologic examination confirmed bilateral optic disc edema and peripheral retinal hemorrhages.

Between May and June 2024, laboratory tests revealed anemia with hemoglobin level of 112 g/L (reference: 130–150), increased serum creatinine to 136.5 µmol/L (estimated glomerular filtration rate [eGFR] by CKD-EPI: 58 mL/min/1.73 m^2^), elevated C-reactive protein (CRP) to 148.21 mg/L (0–5), ferritin to 450 µg/L (20–300), and decreased serum iron to 7.1 µmol/L (11–28) (see Table 2). A single outpatient intravenous infusion of 500 mg ferric carboxymaltose was administered. Thyroid-stimulating hormone was 0.72 µIU/mL (0.35–4.96). Ultrasound examination of the kidneys revealed normal size and parenchymal thickness with parapelvic cysts. Ambulatory blood pressure monitoring (ABPM) showed average values of 140/90 mmHg. Brain MRI revealed a few small supratentorial foci in the frontal and parietal lobes, likely of vascular or residual origin; early signs of an “empty sella” (reduction in pituitary height with herniation of the suprasellar cistern into the sella turcica); a pineal cyst up to 0.5 × 0.6 cm; no cerebrospinal fluid flow disturbances.

**Table 2:** Dynamics of laboratory parameters from May 2024 to January 2025

Parameter, units	05/2024	06/2024	07/2024	08/2024	09/2024	10/2024	12/2024	01/2025	Reference Range
Hemoglobin, g/L	112		108	114	123	141	126	131	130–150
MCV, fL						83	89.1	85.9	80–99
MCH, pg							28.3	28.9	27–31
Leukocytes, × 10^9^/L			7.53			5.9	7.97	6.69	4–9
Platelets, × 10^9^/L			350			327	331	224	160–370
ESR, mm/h			40			40	19		0–15
Ferritin, µg/L	450		303.5		385				20–306
Serum Iron, µmol/L			7.1		8.6				8.1–28.3
Creatinine, µmol/L		136.5	123.9	142.6	148.6	200	184.3	155.5	62–115
eGFR (CKD-EPI), mL/min/1.73 m^2^		58	65	55	52	36	41	49	
Urea, mmol/L		5.46	7.43	8.3			9.12	6.5	2.8–7.2
C-reactive protein, mg/L	58.91	148.21	77.72	90.2	51.1	49.2	69.1	19.06	0–5
Total bilirubin, µmol/L	6.6		6.2			11.2		7.6	2–21
Direct bilirubin, µmol/L						1.9			0–5
Alkaline phosphatase, U/L					97.4	106			30–120
Urine specific gravity			1007				1004	1012	1008–1025
Urine pH			5.0				7.0	7.0	5.0–7.0
Urine sediment			No				No	No	
Urine protein			0				0	0	0–0.15

In August 2024, the patient developed lower leg and foot edema, papular eruptions on the chest and face, and isolated small papules on the lower legs. Fungal cultures from the scalp and inguinal folds were negative. Abdominal ultrasound showed signs of hepatic steatosis, gallbladder angulation, and diffuse pancreatic heterogeneity. Esophagogastroduodenoscopy revealed erosive gastritis and superficial bulbitis, not associated with *Helicobacter pylori*. Otolaryngological examination: bloody discharge in the nasal passages, hyperkeratotic masses in the external auditory canals. Cultures from the external auditory canals were negative for bacterial and fungal pathogens. Diagnosis: chronic bilateral external otitis, eczematous form; fibrotic atresia of the external auditory canals; subatrophic rhinitis. Chest radiography showed no pathology. Findings included elbow joint osteoarthritis, thoracic spine scoliosis, bilateral coxarthrosis (stage II, Kellgren–Lawrence), osteoarthritis of both sacroiliac joints, and early degenerative-dystrophic changes in the pubic symphysis.

In September 2024, nephrology consultation was performed. Given the involvement of the optic nerves, ENT organs, kidneys, and markedly elevated systemic inflammation markers, systemic vasculitis was suspected (with involvement of kidneys, eyes, and ENT structures). Laboratory testing: Rheumatoid factor, ANCA (c-ANCA, p-ANCA), antinuclear factor, aquaporin-4 antibodies, and complement component C3 were within reference ranges. C4 complement component was elevated at 0.44 g/L (0.1–0.4). Prostate-specific antigen and carcinoembryonic antigen levels were within reference ranges.

In October 2024, new papular eruptions appeared on the inner surfaces of the lower limbs.

**Fig. 2:**
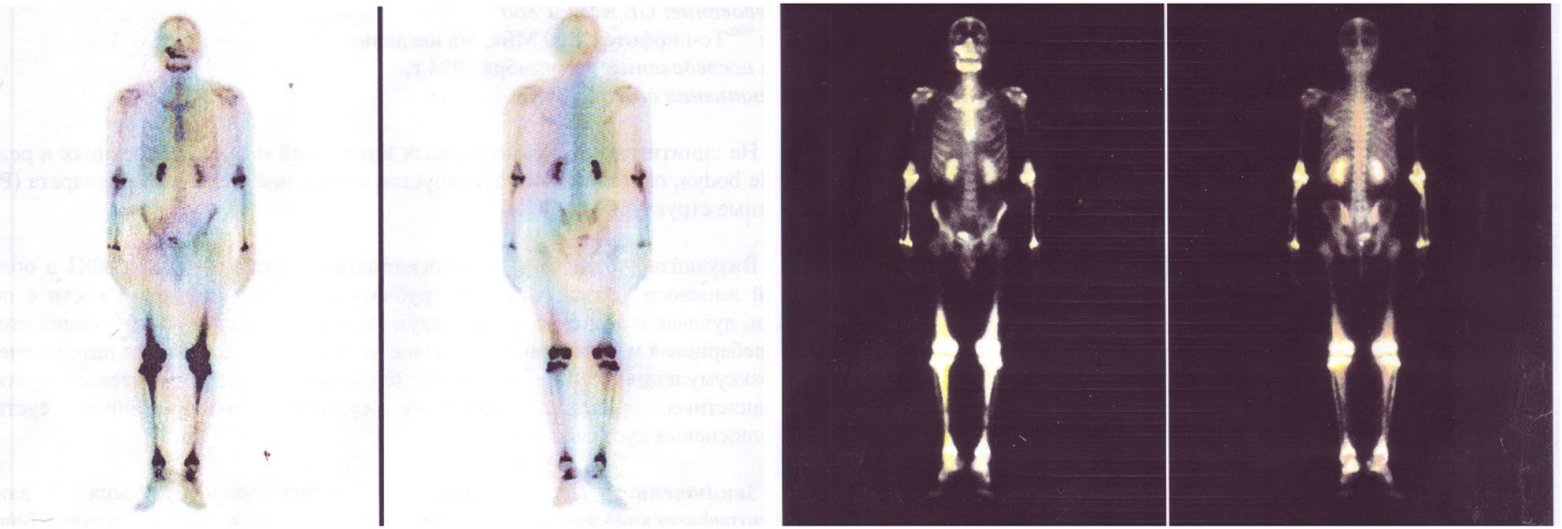
Bone scintigraphy

In November 2024, X-rays of long bones revealed symmetrical mottled intramedullary osteosclerosis with relatively preserved epiphyses, cortical thickening – most prominent in the distal femoral diaphysis – with undulating cortical contours. These findings are characteristic of ECD. Bone scintigraphy showed pathological radiotracer uptake in the facial bones and long tubular bones (humerus, radius, ulna, femur, tibia, fibula bilaterally), as well as in both shoulder, elbow, wrist, knee, ankle, and tarsal joints – findings most consistent with ECD (Fig. 2).

Repeat ophthalmologic evaluation demonstrated optic disc edema and peripheral retinal dystrophy in both eyes, interpreted as further manifestations of ECD disease.

Abdominal and retroperitoneal MRI with contrast revealed bilateral hydronephrosis, left adrenal hyperplasia, and focal bone marrow lesions in the scanned regions.

Renal ultrasound showed bilateral dilation of the pelvicalyceal systems: calyces up to 14 mm, left renal pelvis up to 28 mm, right up to 24 mm. Renal sinus cysts (11 mm and 12 mm) in the left kidney, diffuse changes in renal echotexture, and bilateral calicopyeloectasia were noted.

**Fig. 3:**
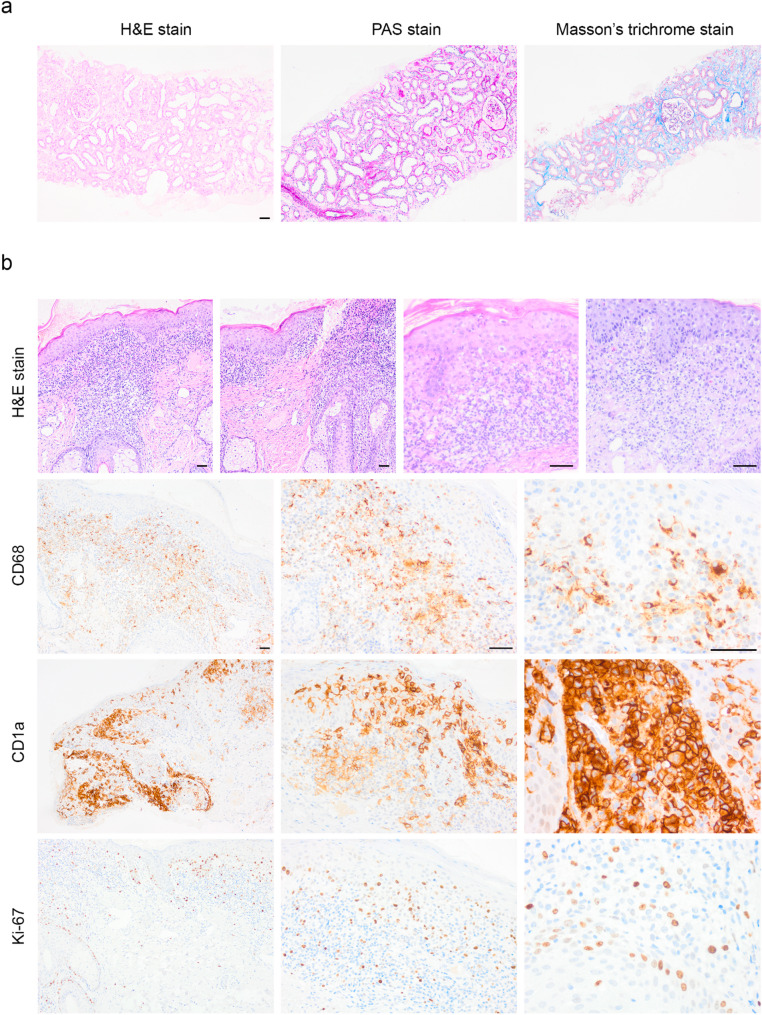
Morphological and immunohistochemical characteristics of lesions in Langerhans cell histiocytosis. **a** – Kidney biopsy: multifocal mononuclear cell infiltration in the cortical interstitium and perinephric fat tissue without signs of glomerulopathy. **b** – Skin biopsy: dermal infiltrate composed of medium-sized cells with bean-shaped nuclei surrounded by eosinophils. Infiltrating cells show strong membranous expression of CD1a and weak nuclear-cytoplasmic Ki-67 expression (6%), confirming the diagnosis of Langerhans cell histiocytosis. CD68 demonstrates diffuse and perinuclear dot-like positivity. Abbreviations: H&E – hematoxylin and eosin; PAS – Periodic acid–Schiff. Scale bar: 100 μm

On November 19, 2024, a renal biopsy was performed (Fig. 3A):

Light microscopy: The sample included a small cortical fragment and perinephric fat. Of the 5 glomeruli, none were sclerotic; one appeared ischemic. Glomeruli were of normal size, without mesangial or endocapillary hypercellularity. Basement membranes were thin and single-contoured. Interstitial fibrosis and tubular atrophy were present but not quantifiable due to limited cortical representation (approx. 20–30% of sample area). Multifocal mild mononuclear interstitial infiltrates without tubulitis. Small arteries appeared unremarkable; no large-caliber arteries were present. Mild arteriolar hyalinosis. Moderate multifocal mononuclear infiltration in perinephric fat. Conclusion: Interstitial fibrosis and tubular atrophy (grade not determined).

Immunofluorescence (IF): IgG, IgM, IgA, C3c, C1q, kappa, lambda, fibrinogen – all negative.

IHC: CD138, IgG, IgG4 – negative.

On December 24, 2024, a scalp skin biopsy was performed (Fig. 3B):

Light microscopy: Sections of skin with “crusts”, epidermis showed parakeratosis, focal acanthosis, and spongiosis. The dermis (papillary and reticular layers) contained an infiltrate of medium-sized cells with grooved/folded nuclei and moderately pale cytoplasm, among eosinophilic granulocytes and lymphoplasmacytic infiltration. The infiltrating cells expressed CD1a (membranous pattern), Langerin (fine granular cytoplasmic pattern), S100 (nuclear and cytoplasmic staining), CD68 (diffuse and perinuclear dot-like positivity). Ki-67 proliferation index: 6%. Negative for CD21. Rare cells expressed ERK-1 (very weak nuclear/cytoplasmic staining). Conclusion: Histopathological features consistent with LCH.

In December 2024, the patient began treatment with trametinib 0.1 mg once daily and desmopressin 0.1 mg twice daily, as prescribed by the attending physician.

In February 2025, the patient consulted a dermatologist with complaints of pink-colored rashes on the cheeks and nasolabial folds, chest, and scalp; periodic edema of the feet and lower legs; erythematous-papular eruptions and scaling on the lower legs. He associated the facial and chest rashes with trametinib intake.

**Fig. 4:**
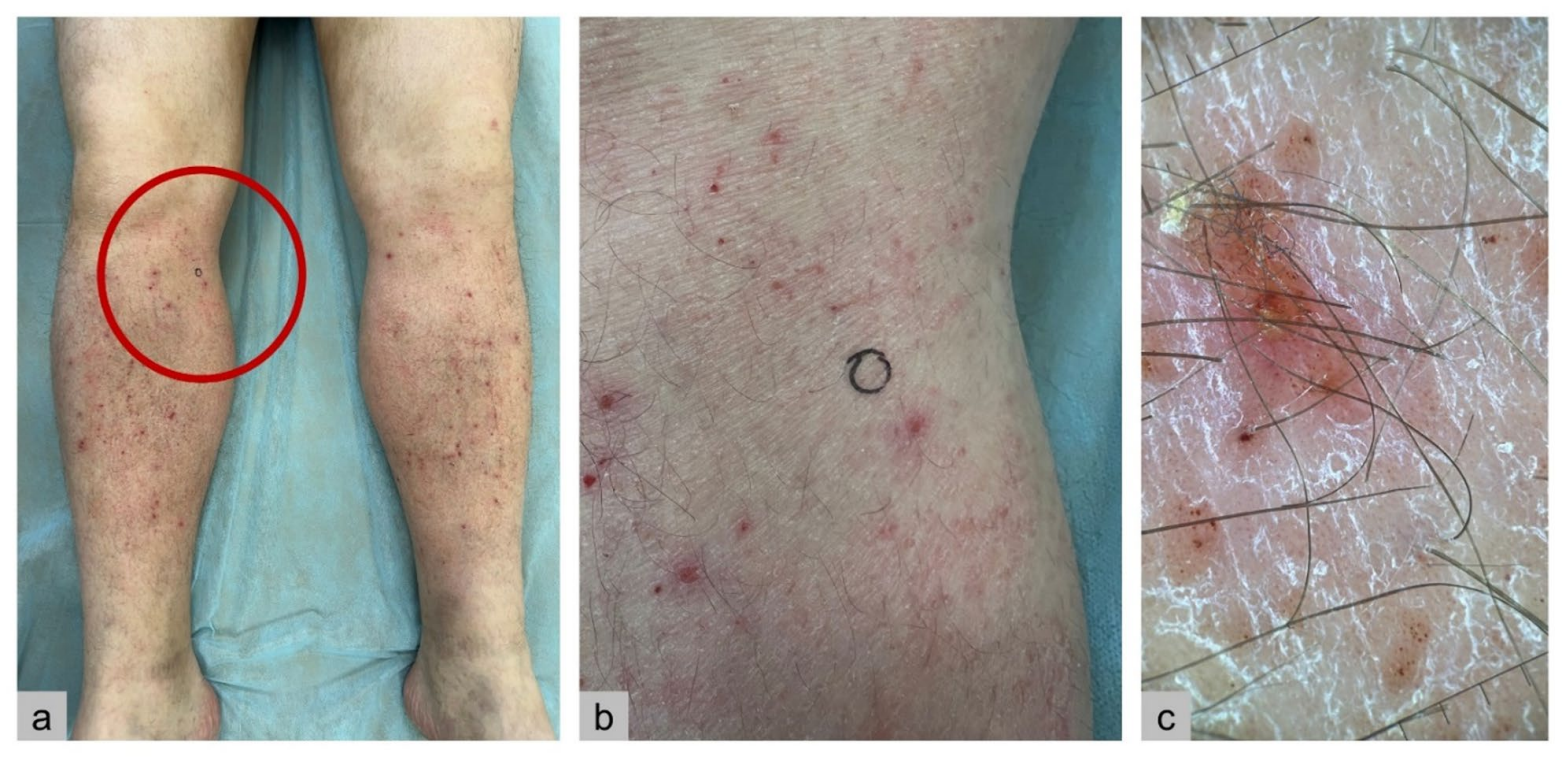
**a** – Macroscopic image of the patient’s lower legs: multiple symmetrical papular eruptions and excoriations. The red circle marks the punch biopsy site. **b** – Macroscopic image of the right lower leg with the punch biopsy site marked. **c** – Dermatoscopic image of the rash on the right lower leg at the biopsy site. The image was taken using an optical adapter (Handyscope, Germany) compatible with a smartphone, 20× magnification

Status localis: erythematous papulopustular eruptions with crusting on the scalp; isolated erythematous-papular lesions on the face, lower legs, and sternum. In the inguinal folds, erythema and maceration were observed (Fig. 4). Visible mucous membranes appeared pink and unremarkable.

Electrocardiogram (February 2025): sinus rhythm, regular, heart rate 69 bpm. Normal electrical axis. QT interval 0.41 s, QTc 0.443 s.

Echocardiography (February 2025): left ventricular hypertrophy – interventricular septal thickness 1.15 cm (ref: 0.6–0.9 cm), posterior wall thickness 1.03 cm (ref: 0.6–0.9 cm), LV mass index 136 g/m^2^. Grade II diastolic dysfunction, moderate left ventricular dilatation. Left ventricular ejection fraction 50%. No regional wall motion abnormalities. Mild mitral regurgitation (Grade I), mild aortic regurgitation (Grade I), mild right ventricular dilatation, tricuspid regurgitation (Grade I–II), pulmonary regurgitation (Grade II).

Status praesens: On physical examination, the general condition was satisfactory. Height 170 cm, weight 87 kg, BMI 28.7 kg/m^2^. Skin was of normal color and moisture. Nasal breathing was free. Respiratory rate 16/min. Percussion: clear pulmonary sound. Auscultation: vesicular breath sounds, no rales. Heart sounds were muffled, rhythm regular, no murmurs. HR 70 bpm. BP 160/120 mmHg. SpO_2_ 97%. Peripheral pulses were palpable and of satisfactory quality. Tongue moist, not coated. Abdomen soft, painless, no signs of peritoneal irritation. Liver at the costal margin. Spleen not palpable. Renal percussion tenderness negative bilaterally.

Clinical diagnosis, based on history, investigations, and examination:

Primary disease: MH: LCH + ECD (associated with *BRAF* V600E mutation), with involvement of: kidneys (perinephric fibrosis, calicopyeloectasia with impaired nitrogen excretion; histologically: interstitial fibrosis, tubular atrophy), skin and mucous membranes (erythematous and ulcerative lesions), CNS (diabetes insipidus, optic disc edema), ocular system (peripheral retinal dystrophy OU), long bones (osteosclerosis and osteolysis in distal segments of humeral and femoral bones), joints (shoulders, elbows, wrists, knees, ankles, tarsal joints), bone marrow, non-specific manifestations (acute-phase inflammatory response, asthenia).

Comorbidities: chronic hypochromic microcytic anemia associated with *STEAP3* (chr2:119247677 A > T); type 4 hereditary hemochromatosis (*SLC40A1*, chr2:189572901 A > T; heterozygous, resulting in amino acid substitution p.Met111Lys, NM_014585.6); chronic bilateral eczematous otitis externa, fibrotic atresia of external auditory canals; moderate myopia, myopic astigmatism.

The patient is under multidisciplinary follow-up and continues treatment with trametinib 1 mg orally once daily and desmopressin 0.1 mg twice daily. For skin lesions, topical therapy is used as needed: metronidazole gel and betamethasone cream. Over a 6-month period, partial regression of scalp and inguinal rashes, as well as improvement in acute-phase markers and a downward trend in serum creatinine, have been observed (Fig. 1c).

**Fig. 5:**
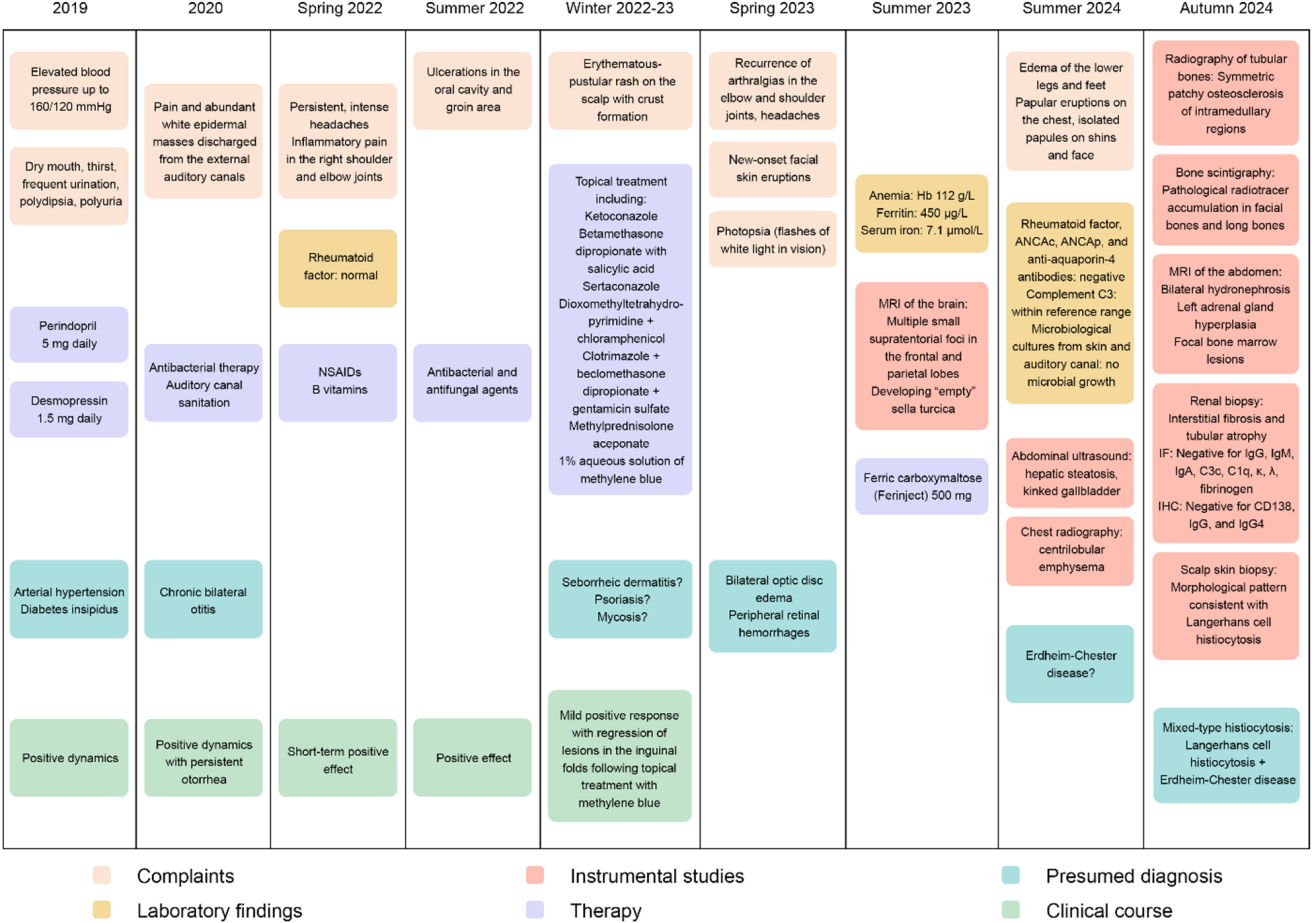
Timeline of patient N. findings and management (2019–2024)

A visual timeline of key diagnostic, therapeutic, and monitoring events is shown in Fig. 5.

## Materials and methods

### Genomic DNA extraction and quality control

The skin sample was minced using sterile scalpels. The minced tissue was then transferred to a lysis mixture containing 180 µL of ATL buffer and 20 µL of Proteinase K. The sample was incubated overnight at 56 °C in a thermoshaker at 1500 rpm. Following incubation, 200 µL of AL buffer and 200 µL of 96% ethanol were added to the lysate, and the mixture was loaded onto a DNA-binding spin column. DNA purification was carried out using AW1 and AW2 wash buffers according to the manufacturer’s protocol (Qiagen, Hilden, Germany). After the final wash and drying step, DNA was eluted in 50 µL of AE buffer. The quality of the extracted DNA was assessed by measuring the concentration using the Qubit dsDNA HS Assay Kit (Biodynami, Huntsville, AL, USA), which yielded a concentration of 33.5 ng/µL. DNA integrity was evaluated by electrophoresis on a 1.5% agarose gel (Qiagen, Hilden, Germany).

For the blood sample, genomic DNA was extracted using standard protocols and subjected to downstream library preparation.

### Library preparation, exome capture and sequencing

Ultrasonic fragmentation was performed with Covaris S220 (Covaris, Inc., Woburn, MA, USA). For the skin sample, library preparation was conducted using VAHTS Universal DNA Library Prep Kit for MGI (Vazyme, China). Blood DNA libraries were prepared using the MGIEasy Universal DNA Library Prep Set (MGI Tech, Shenzhen, China). Enrichment of exonic sequences was achieved using Agilent All Exon v8 (Agilent Technologies, Santa Clara, CA, USA) [26]. Sequencing of protein-coding regions was performed by paired-end sequencing (PE100) on the G-400 (MGI Tech, Shenzhen, China) platform, achieving 100× mean coverage.

### Bioinformatic processing and variant analysis

Raw sequence data quality was evaluated using FastQC v0.11.9 [27]. Sequencing quality reports identified imbalanced bases at read starts, which were trimmed using BBDuk (BBMap v38.96) [28]. Processed reads were then aligned to the GRCh38.p14 reference genome assembly employing bwa-mem2 v2.2.1 [29], with subsequent file format conversion and coordinate sorting implemented through SAMtools v1.9 [30]. Duplicates were marked using Picard v2.22.4 [31]. Variant calling was executed through a dual approach incorporating bcftools mpileup v1.9 [32] and DeepVariant v1.5.0 [33], followed by variant decomposition into biallelic representations using vt decompose v0.5772 [34] and normalization via vt normalize v0.5772. Filtering criteria included coverage depth ≥ 3 and FILTER=PASS for DeepVariant. The resultant VCF files were merged using bcftools-1.9 and annotated via ANNOVAR [35]. The lower threshold for variant allele frequency (VAF) was set at 5%. Clinical interpretation followed ACMG guidelines [36], incorporating minimum coverage thresholds (14×) and population frequency cutoffs (gnomAD v4.1.0 < 1%), with additional consideration of Russian-specific allele frequencies obtained from the RUSeq browser and the Database of Population Frequencies [37–39]. Somatic variant analysis was performed using MuTect2 with default parameters [40].

### Validation of BRAF V600E mutation

Validation was performed to assess the presence of *BRAF* V600E mutation in the NGS *BRAF* V600E positive sample using EntroGen’s BRAF-RT64 kit (EntroGen, Woodland Hills, CA, USA). A PCR-based assay uses allele-specific probes was performed to identify the presence of the *BRAF* V600E, V600K, V600D, V600R, V600M and V600G mutations.

The testing procedure involves three steps: (1) Isolation of DNA from blood sample. (2) Amplification of regions of the *BRAF* gene using allele-specific probes. (3) Detection of amplification product on a Real-Time PCR instrument CFX 96 (BioRad, Saint Petersburg, Russia).

EntroGen’s *BRAF* Codon 600 mutation analysis kits (BRAFX-RT64) are available for research (RUO) and diagnostic (CE-IVD) purposes. It is highly sensitive and specific to detect as little as 1% mutation load. According to the manufacturer’s instructions, the difference between the Ct values of the wild and mutant allele less than 11 confirms the presence of a mutation in the sample being tested.

### Histopathological examination

Biopsies were obtained from the scalp skin with erythematous-desquamative rash (0.7 × 0.2 × 0.2 cm), bone marrow (1.1 × 0.3 × 0.3 cm), and kidney (1.0 × 0.3 × 0.2 cm). Standard histological processing was performed, with paraffin embedding of all samples. Kidney biopsy sections were additionally stained using PAS and Masson’s trichrome methods. IHC was performed on the VENTANA BenchMark ULTRA platform (Roche Diagnostics, Rotkreuz, Switzerland) using the following primary antibodies: CD138, IgG, and IgG4 (for kidney samples); CD1a, CD21, CD68, Ki-67, Langerin, S-100, and ERK-1 (for skin biopsy). All antibodies were used at standard dilutions recommended by the manufacturers. For kidney biopsy samples, direct IF analysis was carried out using antibodies against IgG, IgA, IgM, C3c, C1q, kappa, lambda, and fibrinogen. For bone marrow samples, IF staining targeted CD4, CD8, CD34, CD68, tryptase, S-100, vimentin, and CD11b [41]. The following secondary antibodies were used: Goat Anti-Mouse IgG H&L conjugated with Alexa Fluor^®^ 488 (ab150113), 555 (ab150114), and 647 (ab150115), all from Abcam (Cambridge, MA, USA). Additionally, OPAL650 fluorophore (Akoya Biosciences, Marlborough, MA, USA) was used for multiplex detection. Nuclei were counterstained with Fluoroshield Mounting Medium containing DAPI (ab104139, Abcam). Brightfield microscopy was performed using an Olympus CX43 microscope equipped with an Olympus SC50 camera (Olympus Co., Tokyo, Japan). Multiplex IF imaging was performed using the Mantra 2 Quantitative Pathology Imaging System (Akoya Biosciences) based on an Olympus BX43 microscope, equipped with a scientific-grade multispectral 12-bit monochrome CCD camera and a liquid crystal tunable filter. Quantification and analysis of bone marrow cell populations were performed using QuPath v0.5.1 software.

## Results

### Genetic analysis results

**Fig. 6:**
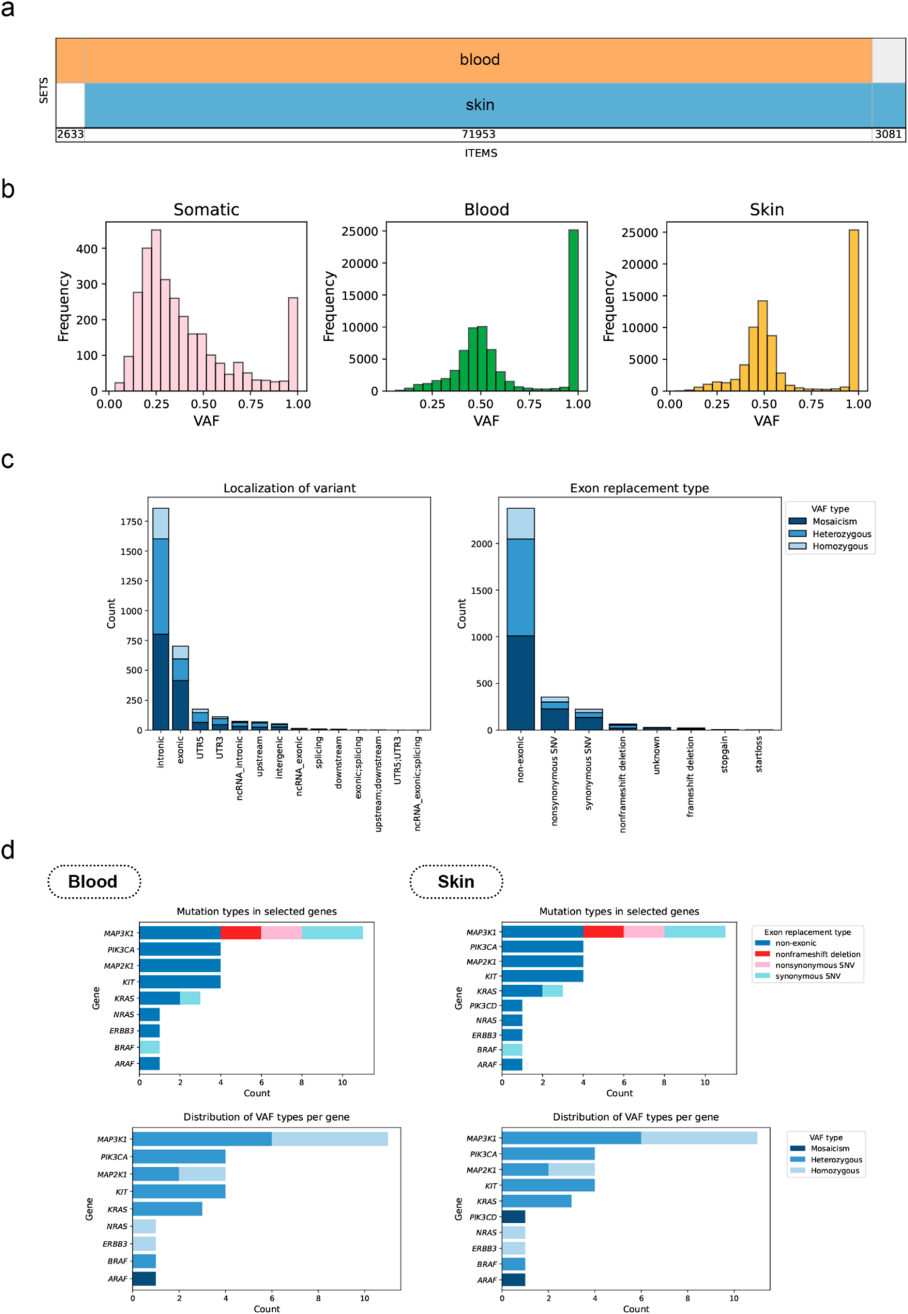
Comparative analysis of somatic (skin only) and germline (blood) variants in a patient with histiocytosis (VAF > 5%). Only reference transcripts were considered. **a** – Venn diagram showing variant overlap between tissues. **b** – Distribution of VAF values. **c** – Variant localization and type distribution. **d** – Mutation frequency across candidate histiocytosis-associated genes. Abbreviation: VAF – variant allele frequency

Comparison of paired blood–skin biopsy samples revealed 71,953 shared variants, with 3,081 unique to the skin (Table S1) and 2,633 germline variants unique to the blood (Fig. 6A). Annotation of somatic skin variants showed VAF ranging from 0.032 to 1.000 (median = 0.318, IQR = 0.278). The distribution was right-skewed (skewness = 1.170), indicating a predominance of low-VAF variants (Fig. 6B). In the blood sample, VAF ranged from 0.061 to 1.000 (median = 0.539, IQR = 0.549), with a near-symmetric distribution (skewness = 0.242). A similar pattern was observed for all skin cell mutations: VAF range 0.032–1.000 (median = 0.527, IQR = 0.535), with a slightly positively skewed distribution (skewness = 0.298). All three variant types were predominantly associated with non-exonic substitutions, especially heterozygous and homozygous ones (> 80%) (Fig. 6C). Nonframeshift deletions predominated among heterozygous variants (52.31%), while nonsynonymous (63.94%), synonymous (60.71%), and variants of unknown significance (80%) were more common among mosaic variants. The greatest number of mutations were detected in *MUC3A*, *HLA-DRB5*, *PRSS2*, *TCAF2*, *MUC2*, *HLA-DRB1*, *FCGBP*, *GOLGA6L2*, *PRSS3*; however, these are regions known to be problematic in next-generation sequencing (NGS) and are not considered pathogenetically relevant. Therefore, only genes from Osipova et al. [21] were included in further analysis. All variants with VAF > 5% were benign (Fig. 6D). Additionally, a *PIK3CD* variant at chr1:9721087G > C was found only in the skin (VAF = 25%) and is likely a sequencing artifact.

**Fig. 7:**
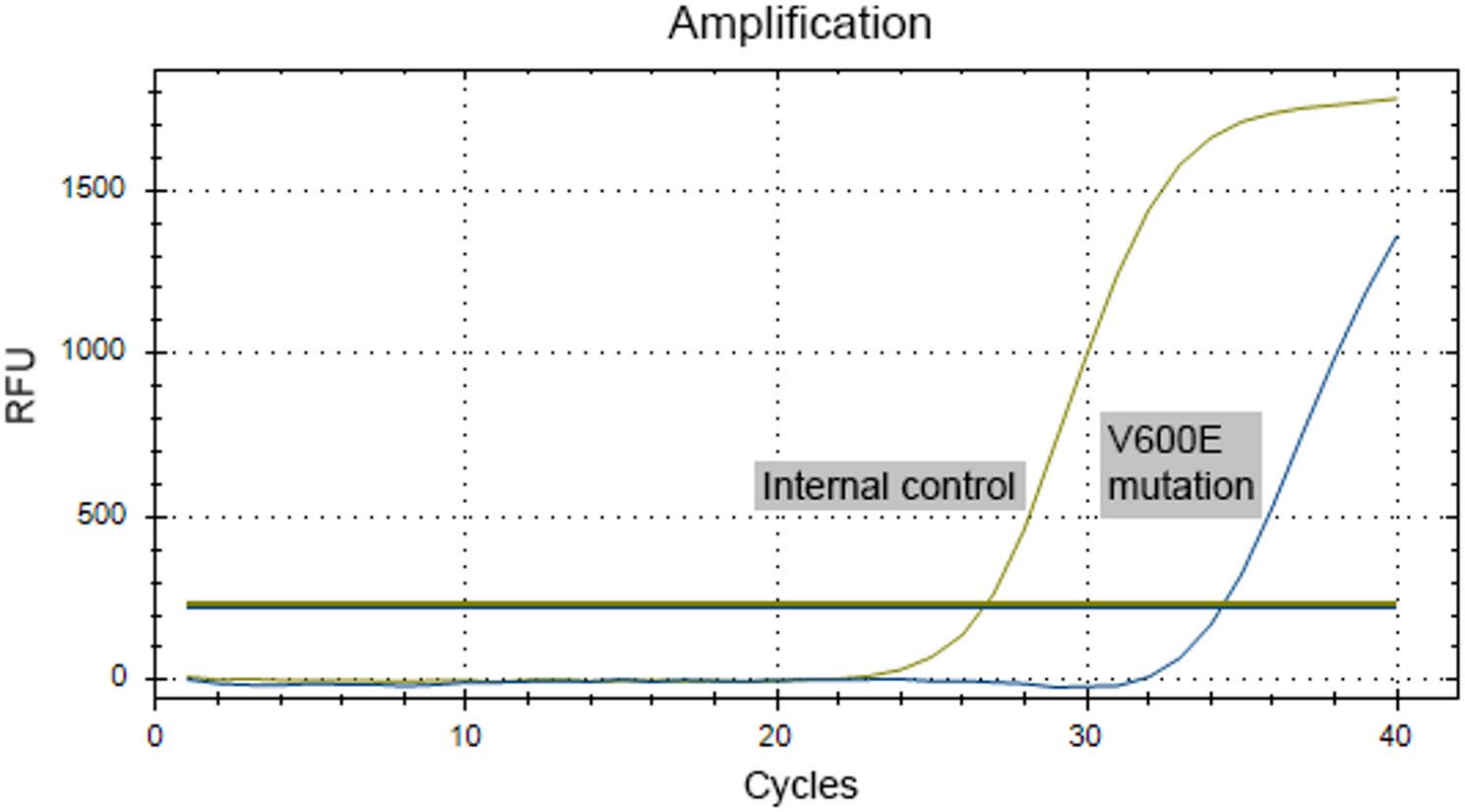
Amplification curve of *BRAF* V600E mutation detection. C_t_ (Internal control) = 27. C_t_ (V600E mutation) = 34.3. Positive for a mutation if: C_t_ (V600E mutation) – C_t_ (Internal control) = 7.3 < 11

Skin biopsy specimens were analyzed for known histiocytosis-related mutations [21, 42]. A *BRAF* V600E variant (VAF 1%; 3/251 reads) was detected in the skin but not in blood and was subsequently validated (Fig. 7). Additionally, a pathogenic mosaic variant *NRAS*(NM_002524.5):c.181 C > G (p.Gln61Glu) was present at low VAF in both blood (1/105 reads) and skin (3/199 reads); this missense variant has previously been reported in a single Russian histiocytosis patient [21].

Whole exome sequencing performed on May 5, 2025, identified a novel intronic variant in *STEAP3* (chr2:119247677 A > T) in heterozygous state, disrupting a splice site (c.523‑2 A > T, NM_182915.3). Such variants are known causes of hypochromic microcytic anemia type II (OMIM: 615234). This variant is absent in gnomAD and the FMBA Russian variant database. All aggregated in silico prediction tools (BayesDel noAF, BayesDel addAF) classify it as pathogenic.

A previously undescribed heterozygous missense variant was also detected in *SLC40A1* (chr2:189572901 A > T), resulting in a p.Met111Lys amino acid change (c.332T > A; NM_014585.6). Such variants are a known cause of type IV hereditary hemochromatosis (OMIM: 606069). Its allele frequency is 0.000002478 in gnomAD (4 heterozygotes) and 0.00000828 in the FMBA database (2 heterozygotes). All in silico predictors classify this variant as pathogenic (BayesDel noAF, BayesDel addAF, MetaRNN, REVEL, MetaLR, MetaSVM).

A heterozygous variant of uncertain significance in *KCNH2*(NM_000238.4):c.273G > T (p.Glu91Asp) associated with epilepsy [43] was also identified, though the patient exhibited no neurological symptoms. All other clinically annotated variants were benign carrier states.

### Pathomorphological analysis of the trephine biopsy

**Fig. 8:**
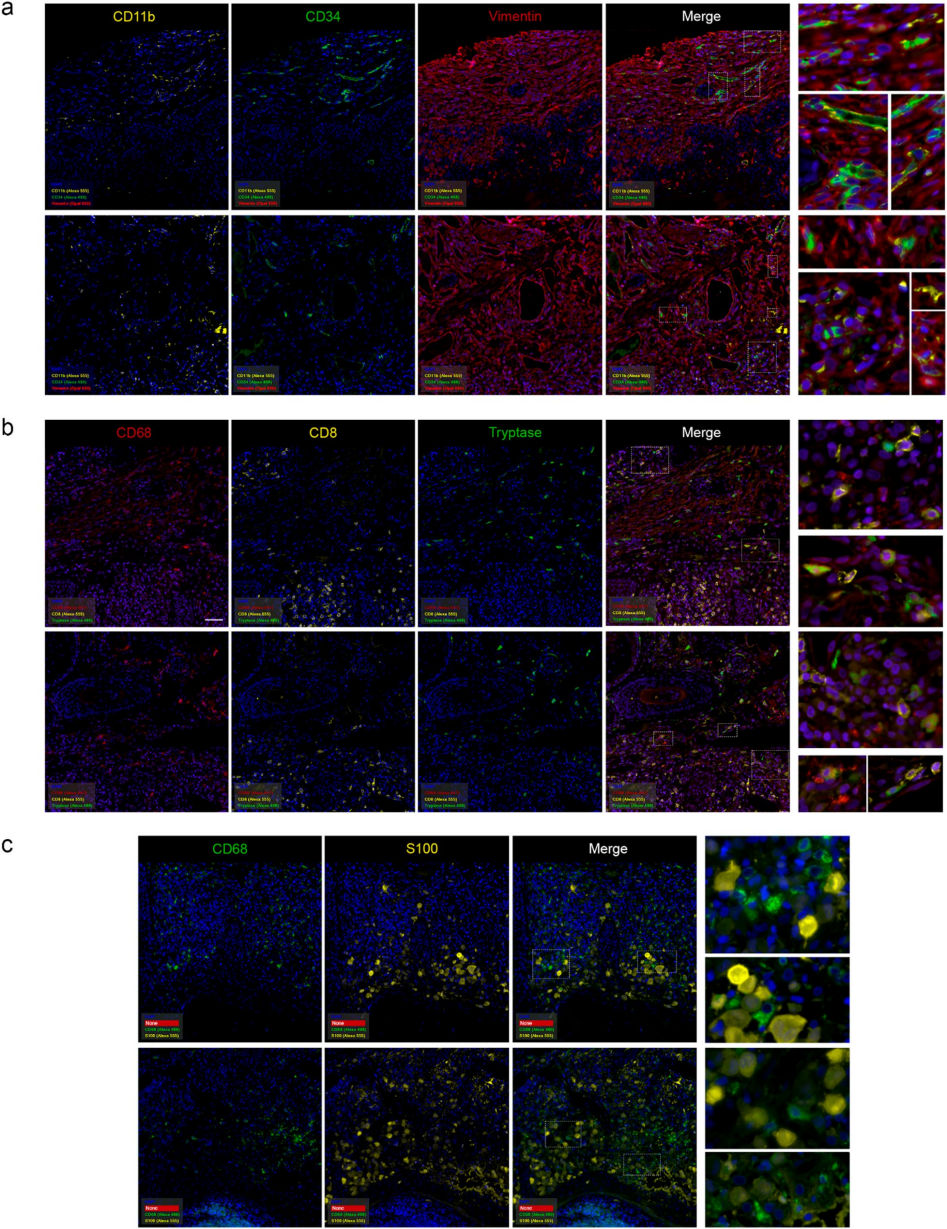
Immunofluorescence analysis of the bone marrow trephine biopsy. **a** – CD11b (yellow), CD34 (green), Vimentin (red). The population of Vimentin^+^CD11b^+^CD34^–^ cells was 11.4 times greater than that of Vimentin^+^CD11b^–^CD34^+^ cells (as a percentage of all Vimentin^+^ cells). **b** – CD8 (yellow), CD68 (red), tryptase (green). Scant Tr^+^ mast cells are shown, which hardly interact with other immunopositive cells. **c** – CD68 (green), S100 (yellow). CD68^+^ cells predominated (~ 56% of immunopositive cells), while CD68^+^S100^+^ cells were less frequent (~ 2%). Two types of CD68 staining were observed: perinuclear, characteristic of Langerhans cells, and diffuse, likely representing cells of the mononuclear phagocyte lineage. Scale bar: 100 μm

IF showed CD34 expression in endothelial cells and fibroblast-like cells (Fig. 8A). Vimentin^+^CD11b^–^CD34^+^ and Vimentin^+^CD11b^+^CD34^–^ cell populations comprised 3 [1.25–20] (median = 0.6814% of Vimentin-positive cells) and 15 [0–56.25] (median  7.778%) cells per high-power field (HPF), respectively (excluding endothelial cells).

Analysis of bone marrow IF images stained for CD8, CD68, and tryptase (Fig. 8B) revealed a density of Tr^+^CD68^–^ cells of 0.02596 [0.01536–0.1915] per 1000 µm^2^ and 10.5 [8.25–19.75] per HPF (356 371.896 µm^2^), corresponding to median = 0.75% of all cells. CD8^+^ cell counts were 89.5 [29.25–131.3] (median 5.945%), CD68^+^ cells 41 [23.75–57.25] (median 2.953%), and Tr^+^CD68^+^ cells 19 [15.25–20.0] (median 1.506%). Contacts (< 1 μm) were observed between 2 mast cells in two HPFs and 3 mast cells in another HPF. The number of Tr^+^CD68^–^ cells contacting CD8^+^ cells was 0.5 [0–3], and that of Tr^+^CD68^+^ cells – 2.5 [2–4].

Analysis of CD68 and S100 markers demonstrated predominance of CD68^+^ macrophage-lineage cells, amounting to 117.5 [33.5–250] per HPF (median = 55.61% of all immunopositive cells), followed by S100^+^ cells – 110 [57–137.5] (median 43.02%) (Fig. 8C). Their median area was 38.75 µm^2^ [24.50–60.00], with a minimum of 8.75 µm^2^ and a maximum of 240.3 µm^2^ (skewness = 1.717). CD68^+^S100^+^ cells were less frequent, 4 [1.5–6.25] per HPF (median 2.174%). Notably, two types of CD68^+^ staining were observed: perinuclear dot-like, typical for LCH cells, and diffuse staining in smaller-volume cells, most likely monocytes or macrophages.

## Discussion

Both ECD and LCH share the somatic activating mutation *BRAF* V600E, found in approximately 50% of cases, suggesting overlap in pathogenetic pathways and explaining their coexistence in MH [44].

According to the 2022 LCH classification [22], the patient fits the multisystem subtype, with involvement of > 2 organs: bones, skin/mucosa, kidneys, endocrine system, CNS, eyes, and bone marrow. Within the framework of MH classification, this presentation most closely corresponds to Type-1 MH (sensu stricto), characterized by the coexistence of LCH and non-Langerhans histiocytosis, most commonly ECD [3].

CNS involvement manifested as diabetes insipidus via pituitary insufficiency and MRI-confirmed pituitary atrophy. Zhou et al. described a patient with isolated pituitary LCH presenting with polydipsia, polyuria, and MRI pituitary stalk thickening [45]. In our case, the pituitary insufficiency may be tied to the primary diagnosis.

No hepatosplenomegaly or cholestatic liver failure characteristic of LCH [22] was found, though hepatic steatosis was noted. Ear involvement is uncommon in LCH, but our patient had chronic bilateral eczematous otitis externa, fibrotic atresia of the external auditory canals, and subatrophic rhinitis. Savina et al. reported a similar chronic otitis case attributed to LCH [46]. Given the long-standing resistance to conventional therapy and response to trametinib, a connection to LCH is plausible.

According to Pires et al., the age at diagnosis of MH varies widely, ranging from 41 to 74 years [47]. In our case, the diagnosis was established at the age of 34, which is likely due to the early manifestation of the disease (the first symptoms appeared at age 29), as well as a pronounced clinical presentation predominantly involving the skin and bones.

Pulmonary involvement in LCH is relatively common (observed in more than 50% of cases with unifocal LCH). The at-risk group includes males aged 20–40 years. The only proven triggering factor is tobacco smoke – more than 90% of patients with pulmonary LCH are smokers [48]. In our case, although the patient fully met the above criteria, only centrilobular emphysema in the upper lung zones – characteristic of smokers – was detected.

A similar case of MH was described by Tsai et al. in a 34-year-old female patient who presented with ulcerative skin lesions on both thighs, osteosclerosis of the distal tibiae, and involvement of the soft tissues of the chest wall [49]. Our patient exhibited almost identical symptoms; however, in our case, the skin lesions were more prominent on the scalp, whereas in the case described by Tsai et al., ulcerative lesions were mainly observed on the lower extremities.

Treatment of patients with MH is particularly challenging. In pulmonary LCH, smoking cessation is strongly recommended to slow disease progression. Reported treatment regimens include glucocorticoids, interferon-α, systemic chemotherapy, radiation therapy, and in certain cases, cladribine (an immunosuppressive agent) and vemurafenib (a protein kinase inhibitor) [47]. Patient N. is currently receiving therapy with trametinib (an allosteric inhibitor of MAPK 1 and 2) and desmopressin (a hormone analogue related to hypothalamic-pituitary regulation, including gonadotropins and their antagonists).

Histological and IF data in our patient provided a deeper insight into the disease biology. Light microscopy revealed an infiltrate of medium-sized cells with grooved nuclei and pale cytoplasm, displaying CD1a, Langerin, S100, and perinuclear dot-like CD68 expression – classic hallmarks of LCH. However, the additional presence of diffuse CD68^+^ macrophages and weak ERK-1 nuclear staining highlights a pathological spectrum extending beyond conventional LCH morphology. The Ki-67 index of 6% indicated low proliferative activity, consistent with a smoldering inflammatory process rather than an aggressive neoplasm.

IF analysis further revealed a structured yet functionally imbalanced microenvironment. The low prevalence of CD34^+^ cells argues against a blast-driven process, while the relative enrichment of CD11b^+^ elements is more consistent with chronic inflammatory infiltration than with neoplastic proliferation. Mast cells (~ 2.26% of all cells) were sparse and rarely engaged in close contacts with other immune populations, suggesting a limited regulatory role. CD8^+^ T lymphocytes were present but modest in proportion (~ 6% of all cells), while the predominance of CD68^+^ macrophages created a pro-inflammatory milieu that was nonetheless insufficient to eliminate pathological clones, yet capable of sustaining tissue injury. Of particular note, the coexistence of perinuclear-dot CD68^+^ histiocytes with diffusely CD68^+^ macrophages reflects both canonical LCH cells and activated myeloid infiltrates, bridging the histological features of LCH and ECD within one tissue context. Thus, the histology and IF together illustrate a “hybrid” lesion in which classical dendritic LCH cells are embedded in an abnormal macrophage-rich stroma, providing a morphological correlate of the MH phenotype.

This microenvironment suggests cellular interplay, the origin of these macrophages cannot be determined from a single-timepoint analysis. Recent pediatric studies have proposed that macrophage-like histiocytes may transdifferentiate into Langerhans-like cells [50]; however, in our adult-onset patient with established mixed LCH/ECD, this remains a purely hypothetical explanation, which would require single-cell or longitudinal analyses to test.

Conceptual frameworks in LCH research emphasize that the differentiation stage of the first cell acquiring a MAPK pathway-activating mutation strongly shapes disease phenotype, organ involvement, and outcomes [51]. Mutations in hematopoietic stem/progenitor cells or multipotent progenitors can lead to multisystem disease with widespread tissue distribution, whereas mutations in more differentiated myeloid precursors result in more limited presentations. Circulating BRAF- or KRAS-mutant myeloid cells derived from these progenitors can give rise to LCs (CD207^+^/CD1a^+^) and may infiltrate the CNS, contributing to neurodegeneration.

The molecular and cellular mechanisms underlying the pathogenesis of LCH are associated with impaired differentiation and function of antigen-presenting cells. Specifically, elevated levels of CD34^+^ cells have been identified in the blood of LCH patients; these cells are capable of differentiating into LC-like dendritic cells (CD1a^+^CD83^+^Lag^+^), a subset of which express CD2 and exhibit reduced viability in vitro. Although these cells elicit an allogeneic T-cell response, they often fail to initiate a primary antigen-specific response, suggesting functional defects consistent with hematopoietic progenitor involvement [52]. In addition, large adult ECD cohorts provide a complementary, data-driven perspective: clustering analysis of 661 patients identified three stable groups – “Widespread Disease” (WID), “Limited Disease” (LIM), and “MAP2K1-RDD” (MAP) – based on somatic mutation (*BRAF* V600E vs. *MAP2K1*), organ involvement, and overlap with other histiocytoses [53].

CD34 is a transmembrane sialomucin molecule predominantly expressed on hematopoietic stem/progenitor cells, endothelial cells, and embryonic fibroblasts. It is also present at later stages of hematopoietic development, including pro-T/B cells, monocyte/mast cell progenitors, megakaryocyte colony-forming units (CFU-Mk), and erythroid precursors (BFU-E). Furthermore, CD34 expression is retained in mature NK cells [54]. The precise functions of CD34 remain unclear; it is hypothesized to regulate interactions with the bone marrow microenvironment and control cell proliferation, although data are conflicting and depend on the experimental model used. At present, CD34 is primarily viewed as a marker of functional cellular subsets, without a well-established functional role in hematopoietic stem cells [55].

CD68 is a glycoprotein highly expressed in macrophages and other monocyte-lineage cells. It resides primarily in lysosomes and participates in intracellular functions, including phagocytosis. CD68 expression is minimal in lymphocytes, dendritic cells, non‑mesenchymal cells (fibroblasts, endothelial cells) and tumor cells [56]. CD11b is the α-subunit of the β2-integrin heterodimer that binds ICAM-1, iC3b, and fibrinogen. It plays a crucial role in phagocytosis, adhesion, and the regulation of inflammatory responses. Deficiency of β2-integrin leads to impaired phagocytic function and compromised immune defense. In contrast, CD11b knockout in mice impairs complement-mediated phagocytosis and vasculitis but does not significantly affect neutrophil migration [57]. CD11b is also a key regulator of antitumor immunity, modulating macrophage polarization and shaping the immune microenvironment. Loss of CD11b enhances the immunosuppressive phenotype of tumor-associated macrophages (TAMs), promotes accumulation of FoxP3^+^ regulatory T cells, reduces CD8^+^ T-cell infiltration, and accelerates tumor growth. Pharmacological activation of CD11b by the agonist LA1 repolarizes TAMs toward a pro-inflammatory phenotype, enhances T-cell responses, and suppresses tumor progression. CD11b also contributes to vasculogenesis by regulating vessel maturation and nutrient supply to the tumor [58]. Neither CD68 nor CD11b are characteristic markers of resting LCs; their expression indicates pathological activation in LCH [59]. CD11b expression has been identified in LCH tissue samples from skin, mucosa, bone, and lymph nodes [60]. The active phase of LCH is associated with an increase in CD11b^high^ and CD11b^int/low^ populations, including CD207^+^CD1a^+^ cells, detectable in peripheral blood [61]. In the present case, the detection of LCH/ECD with systemic inflammation, vasculitis-like features, and multiorgan involvement is consistent with pathological activation of CD11b^+^ myeloid cells. This supports the concept that CD11b-driven immune dysregulation may underlie both the chronic inflammatory response and the extensive tissue damage observed in the patient.

Bone marrow involvement occurs in 2–33% of LCH cases, more commonly in infants with multisystem disease. It is associated with anemia, presence of CD1a^+^ histiocytes in bone marrow trephines, and poor prognosis [62].

The patient’s anemia is likely related to the identified *STEAP3* splice-site variant c.523‑2 A > T. A nonsense mutation in the same gene (c.300 C > A, p.Cys100Ter) was described in a Pakistani family with three siblings affected by severe anemia and iron overload [63]. First described in 2011, the disorder also manifests as hypogonadism and elevated serum ferritin levels before transfusions begin. The pathogenesis involves disruption of STEAP3, an endosomal ferrireductase that converts Fe^3+^ to Fe^2+^ in the transferrin-dependent iron uptake pathway in erythroid cells. Impaired ferrireductase activity reduces iron availability for incorporation into protoporphyrin (via ferrochelatase), leading to microcytic hypochromic anemia and compensatory iron accumulation in the reticuloendothelial system (in siderosomes and mitochondria). The disease is presumed to manifest in compound heterozygosity with one null allele and one weakly expressed (allele-specific regulation).

A study of 3,205 healthy Chinese donors demonstrated that *STEAP3* mutations affecting the NAD(P)H-binding motif (p.Arg59Cys) or heme-binding sites (p.His316Asn) significantly impaired ferrireductase function. However, heterozygous carriers, including those with the p.Cys261Ter nonsense mutation, showed no clinical signs of anemia or iron metabolism abnormalities. The mutant mRNA was not fully degraded, and the truncated protein formed aberrant aggregates in endosomes and on the plasma membrane [64]. Our patient also carried the heterozygous *STEAP3*(NM_182915.3):c.-24G > A variant, which is common in gnomAD (including in homozygous individuals; *n* = 126,453), suggesting uncertain pathogenicity. Thus, the roles of c.523‑2 A > T and c.-24G > A in this patient’s clinical presentation require further evaluation.

Knockout of *ANXA10* has been shown to increase TFRC (transferrin receptor) levels by inhibiting its autophagic degradation, leading to intracellular iron (Fe^2+^) accumulation via the TFRC–STEAP3–SLC11A2 axis. Concurrently, expression of *SLC40A1* (ferroportin) is downregulated, reducing iron export and triggering ferroptosis [65]. In addition to iron overload, lipid ROS accumulation is a critical factor in ferroptotic cell death [66, 67].

Ferroportin disease (type IV hereditary hemochromatosis) is the second most common genetic iron overload disorder after HFE-hemochromatosis. It is caused by heterozygous mutations in *SLC40A1*, encoding ferroportin, the only known cellular Fe^2+^ exporter in humans, primarily localized to the basolateral membrane of duodenal enterocytes (less active in the colon) and macrophage membranes. Ferroportin expression and function are regulated by intracellular iron via an iron-responsive element (IRE) in the 5’UTR of its mRNA, which binds iron-responsive proteins (IRPs) [68].

The disease manifests in two major phenotypes. The classical (macrophage) phenotype results from loss-of-function mutations (e.g., Δ162, Δ160–162, G323V, G490D) that impair cellular localization and membrane trafficking, reducing iron export and leading to macrophage iron accumulation. It is associated with high serum ferritin, normal or low transferrin saturation, and may include anemia. The course is typically indolent, complicating early diagnosis. The nonclassical (hepatocellular) phenotype involves gain-of-function mutations resistant to hepcidin inhibition (e.g., N144H, N144T, N144D). Ferroportin remains membrane-bound and exports iron constitutively, resulting in elevated transferrin saturation and hepatic iron deposition, similar to classical hemochromatosis. Ferroportin functions as a multimer; mutant proteins exert a dominant-negative effect on wild-type ferroportin, disrupting iron export regulation [69].

SLC40A1 interacts with JAK2, which in turn interacts with BRAF (co-expression and experimentally determined interaction per STRING [70]). JAK2 is also directly linked to BRAF via RAF1, NRAS, KRAS, and HRAS. Although no direct experimental interaction between SLC40A1 and STEAP3 has been documented, both appear alongside SMAD6 (previously implicated in LCH [13]) in the “Negative regulation of programmed cell death subnetwork” pathway.

## Conclusion

The presented case illustrates a rare overlapping phenotype of LCH and ECD with early-onset, multisystem involvement, and severe anemia. The key pathogenetic driver is the somatic *BRAF* V600E mutation, supporting clonal proliferation of histiocytes. Germline variants in *STEAP3* and *SLC40A1* create a background of iron dysregulation and chronic inflammation, which likely modulates the course and severity of the disease. Morphological and immunophenotypic data confirm the coexistence of true Langerhans-type cells and a dominant macrophage/stromal component, reflecting the mixed pathogenesis. The immune microenvironment is characterized by prevailing innate activation and limited cytotoxic T-cell involvement, favoring persistence of the histiocytic clone. This complex architecture explains the partial response to MAPK-targeted therapy and highlights the need for strategies addressing both clonal and reactive compartments. Timely integration of clinical, pathological, and molecular findings is crucial for accurate diagnosis. Multidisciplinary discussion remains essential for personalized treatment planning and improvement of patient outcomes.

## Electronic Supplementary Material

Below is the link to the electronic supplementary material.

Supplementary Material 1

## Data Availability

Corresponding author upon request.

## References

[1] Pegoraro F, Papo M, Cohen-Aubart F, Peyronel F, Lugli G, Trambusti I, et al. Long-term outcome and prognosis of mixed histiocytosis (Erdheim-Chester disease and Langerhans cell histiocytosis). EClinicalMedicine. 2024;73:102658. 10.1016/j.eclinm.2024.102658. 38841707 10.1016/j.eclinm.2024.102658 PMC11152896

[2] Dai JW, Lin H, Chang L, Li J, Zhou DB, Cao XX. The clinical spectrum and prognostic factors of Erdheim-Chester disease and mixed Langerhans cell histiocytosis and Erdheim-Chester disease. Ann Hematol. 2023;102(12):3335–43. 10.1007/s00277-023-05501-1. 37922006 10.1007/s00277-023-05501-1

[3] Bonometi A, for Associazione Italiana Ricerca Istiocitosi AIRI ONLUS. The triptych of mixed histiocytosis: a systematic review of 105 cases and proposed clinical classification. Leuk Lymphoma. 2021;62(1):32–44. 10.1080/10428194.2020.1824070. 32969291 10.1080/10428194.2020.1824070

[4] Gulati N, Allen CE. Langerhans cell histiocytosis: Version 2021. Hematol Oncol. 2021;39(1):15–23. 10.1002/hon.2857. 34105821 10.1002/hon.2857 PMC9150752

[5] Yoon SO. Pathologic characteristics of histiocytic and dendritic cell neoplasms. Blood Res. 2024;59(1):18. 10.1007/s44313-024-00015-9. 38713245 10.1007/s44313-024-00015-9 PMC11076448

[6] Emile JF, Cohen-Aubart F, Collin M, Fraitag S, Idbaih A, Abdel-Wahab O, et al. Histiocytosis Lancet. 2021;398(10295):157–70. 10.1016/S0140-6736(21)00311-1. 33901419 10.1016/S0140-6736(21)00311-1 PMC9364113

[7] Halbritter F, Farlik M, Schwentner R, Jug G, Fortelny N, Schnöller T, et al. Epigenomics and Single-Cell Sequencing Define a Developmental Hierarchy in Langerhans Cell Histiocytosis. Cancer Discov. 2019;9(10):1406–21. 10.1158/2159-8290.CD-19-0138. 31345789 10.1158/2159-8290.CD-19-0138 PMC6795548

[8] Xiao Y, van Halteren AGS, Lei X, Borst J, Steenwijk E, de Wit T, et al. Bone marrow-derived myeloid progenitors as driver mutation carriers in high- and low-risk Langerhans cell histiocytosis. Blood. 2020;136(19):2188–99. 10.1182/blood.2020005209. 32750121 10.1182/blood.2020005209

[9] Abagnale G, Schwentner R, Ben Soussia-Weiss P, van Midden W, Sturtzel C, Pötschger U, et al. BRAFV600E induces key features of LCH in iPSCs with cell type-specific phenotypes and drug responses. Blood. 2025;145(8):850–65. 10.1182/blood.2024026066. 39630039 10.1182/blood.2024026066 PMC11867135

[10] Rodriguez-Galindo C, Allen CE. Langerhans cell histiocytosis. Blood. 2020;135(16):1319–31. 10.1182/blood.2019000934. 32106306 10.1182/blood.2019000934

[11] Allen CE, Merad M, McClain KL. Langerhans-Cell Histiocytosis. N Engl J Med. 2018;379(9):856–68. 10.1056/NEJMra1607548. 30157397 10.1056/NEJMra1607548 PMC6334777

[12] Cai HC, Chen J, Liu T, Cai H, Duan MH, Li J, et al. Langerhans cell histiocytosis in adolescent patients: a single-centre retrospective study. Orphanet J Rare Dis. 2022;17(1):268. 10.1186/s13023-022-02436-0. 35841042 10.1186/s13023-022-02436-0 PMC9288061

[13] Peckham-Gregory EC, Chakraborty R, Scheurer ME, Belmont JW, Abhyankar H, Sengal AG, et al. A genome-wide association study of LCH identifies a variant in SMAD6 associated with susceptibility. Blood. 2017;130(20):2229–32. 10.1182/blood-2017-08-800565. 28935696 10.1182/blood-2017-08-800565 PMC5691246

[14] Ozer E, Sevinc A, Ince D, Yuzuguldu R, Olgun N. BRAF V600E Mutation: A Significant Biomarker for Prediction of Disease Relapse in Pediatric Langerhans Cell Histiocytosis. Pediatr Dev Pathol. 2019;22(5):449–55. 10.1177/1093526619847859. 31072207 10.1177/1093526619847859

[15] Feng S, Han L, Yue M, Zhong D, Cao J, Guo Y, et al. Frequency detection of BRAF V600E mutation in a cohort of pediatric langerhans cell histiocytosis patients by next-generation sequencing. Orphanet J Rare Dis. 2021;16(1):272. 10.1186/s13023-021-01912-3. 34116682 10.1186/s13023-021-01912-3 PMC8196454

[16] Whitlock JA, Geoerger B, Dunkel IJ, Roughton M, Choi J, Osterloh L, et al. Dabrafenib, alone or in combination with trametinib, in BRAF V600-mutated pediatric Langerhans cell histiocytosis. Blood Adv. 2023;7(15):3806–15. 10.1182/bloodadvances.2022008414. 36884302 10.1182/bloodadvances.2022008414 PMC10393756

[17] Berres ML, Lim KP, Peters T, Price J, Takizawa H, Salmon H, et al. BRAF-V600E expression in precursor versus differentiated dendritic cells defines clinically distinct LCH risk groups [published correction appears in J Exp Med. 2015;212(2):281. 10.1084/jem.2013097701202015c. 10.1084/jem.2013097701202015c PMC4322054 25646268

[18] Chakraborty R, Hampton OA, Shen X, Simko SJ, Shih A, Abhyankar H, et al. Mutually exclusive recurrent somatic mutations in MAP2K1 and BRAF support a central role for ERK activation in LCH pathogenesis. Blood. 2014;124(19):3007–15. 10.1182/blood-2014-05-577825. 25202140 10.1182/blood-2014-05-577825 PMC4224195

[19] Alayed K, Medeiros LJ, Patel KP, Zuo Z, Li S, Verma S, et al. BRAF and MAP2K1 mutations in Langerhans cell histiocytosis: a study of 50 cases. Hum Pathol. 2016;52:61–7. 10.1016/j.humpath.2015.12.029. 26980021 10.1016/j.humpath.2015.12.029

[20] Novosad O, Skrypets T, Pastushenko Y, Titorenko I, Martynchyk A, Skachkova O, et al. MAPK/ERK signal pathway alterations in patients with Langerhans Cell Histiocytosis. Změny v signální dráze MAPK/ERK u pacientů s histiocytózou Langerhansových buněk. Klin Onkol. 2018;31(2):130–6. 10.14735/amko2018130. 29708356 10.14735/amko2018130

[21] Osipova DS, Raykina EV, Kozlova YA, Lyudovskikh EI, Evseev DA, Kalinina II, et al. Clinicogenomic associations in patients with Langerhans cell histiocytosis: a cohort study. Pediatr Hematology/Oncology Immunopathol. 2023;22(4):102–7. 10.24287/1726-1708-2023-22-4-102-107.

[22] Goyal G, Tazi A, Go RS, Rech KL, Picarsic JL, Vassallo R, et al. International expert consensus recommendations for the diagnosis and treatment of Langerhans cell histiocytosis in adults. Blood. 2022;139(17):2601–21. 10.1182/blood.2021014343. 35271698 10.1182/blood.2021014343 PMC11022927

[23] McKinney RA, Wang G. Langerhans Cell Histiocytosis and Other Histiocytic Lesions. Head Neck Pathol. 2025;19(1):26. 10.1007/s12105-025-01766-2. 39998733 10.1007/s12105-025-01766-2 PMC11861498

[24] Emile JF, Abla O, Fraitag S, Horne A, Haroche J, Donadieu J, et al. Histiocyte Society. Revised classification of histiocytoses and neoplasms of the macrophage-dendritic cell lineages. Blood. 2016;127(22):2672–81. 10.1182/blood-2016-01-690636. 26966089 10.1182/blood-2016-01-690636 PMC5161007

[25] Go RS, Jacobsen E, Baiocchi R, Buhtoiarov I, Butler EB, Campbell PK, et al. Histiocytic Neoplasms, version 2.2021. J Natl Compr Canc Netw. 2021;19(11):1277–303. 10.6004/jnccn.2021.0053. 34781268 10.6004/jnccn.2021.0053

[26] Belova V, Pavlova A, Afasizhev R, Moskalenko V, Korzhanova M, Krivoy A, et al. System analysis of the sequencing quality of human whole exome samples on BGI NGS platform. Sci Rep. 2022;12(1):609. 10.1038/s41598-021-04526-8. 35022470 10.1038/s41598-021-04526-8 PMC8755732

[27] Andrews S, FastQC:. A quality control tool for high throughput sequence data. 2010. Available online: http://www.bioinformatics.babraham.ac.uk/projects/fastqc/. Accessed 20 April 2025.

[28] Joint Genome Institute. Available online: https://jgi.doe.gov/data-and-tools/software-tools/bbtools/bb-tools-user-guide/bbduk-guide/ (accessed on 20 April 2025).

[29] Li H, Durbin R. Fast and accurate short read alignment with Burrows—Wheeler transform. Bioinformatics. 2009;25(14):1754–60. 1093/bioinformatics/btp324. 19451168 10.1093/bioinformatics/btp324 PMC2705234

[30] Li H, Handsaker B, Wysoker A, Fennell T, Ruan J, Homer N, et al. 1000 Genome Project Data Processing Subgroup. The Sequence Alignment/Map format and SAMtools. Bioinformatics. 2009;25(16):2078–9. 10.1093/bioinformatics/btp352. 10.1093/bioinformatics/btp352 PMC2723002 19505943

[31] Picard Toolkit. version 2.22.4; Broad Institute: Cambridge, MA, USA. 2019; Available online: https://broadinstitute.github.io/picard/ (accessed on 20 April 2025).

[32] Li H. A statistical framework for SNP calling, mutation discovery, association mapping and population genetical parameter estimation from sequencing data. Bioinformatics. 2011;27(21):2987–93. 10.1093/bioinformatics/btr509. 21903627 10.1093/bioinformatics/btr509 PMC3198575

[33] Poplin R, Chang P-C, Alexander D, Schwartz S, Colthurst T, Ku A, et al. A universal SNP and small-indel variant caller using deep neural networks. Nat Biotechnol. 2018;36(10):983–7. 10.1038/nbt.4235. 30247488 10.1038/nbt.4235

[34] Tan A, Abecasis GR, Kang HM. Unified Representation of Genetic Variants. Bioinformatics. 2015;31(13):2202–4. 10.1093/bioinformatics/btv112. 25701572 10.1093/bioinformatics/btv112 PMC4481842

[35] Wang K, Li M, Hakonarson H, ANNOVAR. Functional annotation of genetic variants from high-throughput sequencing data. Nucleic Acids Res. 2010;38(16):e164. 10.1093/nar/gkq603. 20601685 10.1093/nar/gkq603 PMC2938201

[36] Li Q, Wang K, InterVar. Clinical Interpretation of Genetic Variants by the 2015 ACMG-AMP Guidelines. Am. J Hum Genet. 2017;100(2):267–80. 10.1016/j.ajhg.2017.01.004. 10.1016/j.ajhg.2017.01.004 PMC5294755 28132688

[37] Karczewski KJ, Francioli LC, Tiao G, Cummings BB, Alföldi J, Wang Q, et al. The mutational constraint spectrum quantified from variation in 141,456 humans. Nature. 2020;581(7809):434–43. 10.1038/s41586-020-2308-7. 32461654 10.1038/s41586-020-2308-7 PMC7334197

[38] Barbitoff YA, Khmelkova DN, Pomerantseva EA, Slepchenkov AV, Zubashenko NA, Mironova IV, et al. Expanding the Russian allele frequency reference via cross-laboratory data integration: insights from 7452 exome samples. Natl Sci Rev. 2024;11(10):nwae326. 10.1093/nsr/nwae326. 39498263 10.1093/nsr/nwae326 PMC11533896

[39] Database of Population frequencies of genetic variants of the population of the russian federation. [Internet]. FMBA of Russia. 2024. Available online: https://gdbpop.nir.cspfmba.ru/. Accessed 20 April 2025.

[40] Cibulskis K, Lawrence MS, Carter SL, Sivachenko A, Jaffe D, Sougnez C, et al. Sensitive detection of somatic point mutations in impure and heterogeneous cancer samples. Nat Biotechnol. 2013;31:213–9. 10.1038/nbt.2514. 23396013 10.1038/nbt.2514 PMC3833702

[41] Buchwalow I, Samoilova V, Boecker W, Tiemann M. Multiple immunolabeling with antibodies from the same host species in combination with tyramide signal amplification. Acta Histochem. 2018;120(5):405–11. 10.1016/j.acthis.2018.05.002. 29739626 10.1016/j.acthis.2018.05.002

[42] Jouenne F, Chevret S, Bugnet E, et al. Genetic landscape of adult Langerhans cell histiocytosis with lung involvement. Eur Respir J. 2020;55(2):1901190. 10.1183/13993003.01190-2019. 31806714 10.1183/13993003.01190-2019

[43] Li X, Liu N, Bai R. Variant frequencies of KCNQ1, KCNH2, and SCN5A in a Chinese inherited arrhythmia cohort and other disease cohorts undergoing genetic testing. Ann Hum Genet. 2020;84(2):161–8. 10.1111/ahg.12359. 31696929 10.1111/ahg.12359

[44] Hervier B, Haroche J, Arnaud L, Charlotte F, Donadieu J, Néel A, French Histiocytoses Study Group, et al. Association of both Langerhans cell histiocytosis and Erdheim-Chester disease linked to the BRAFV600E mutation. Blood. 2014;124(7):1119–26. 10.1182/blood-2013-12-543793. 24894769 10.1182/blood-2013-12-543793

[45] Zhou W, Rao J, Li C. Isolated Langerhans cell histiocytosis in the hypothalamic-pituitary region: a case report. BMC Endocr Disord. 2019;19(1):143. 10.1186/s12902-019-0474-0. 31856773 10.1186/s12902-019-0474-0 PMC6924050

[46] Savina VYu, Solovyeva SE, Dolzhansky OV, Metelin AV. Clinical and morphological analysis of Langerhans cell histiocytosis in a child of early age. Pirogov Russian J Surg. 2021;6–2101–4. 10.17116/hirurgia2021062101. (In Russ.). 10.17116/hirurgia2021062101 34032796

[47] Pires Y, Jokerst CE, Panse PM, Kipp BR, Tazelaar HD. Combined Erdheim-Chester Disease and Langerhans Cell Histiocytosis in the Lung: A Report of 2 Patients With Overlap Syndrome. AJSP: Reviews Rep. 2020;25(1):33–9. 10.1097/PCR.0000000000000349.

[48] Potapenko VG, Baykov VV, Zinchenko AV, Potikhonova NA. Langerhans cell histiocytosis in adults: literature review. Onkogematologiya = Oncohematology. 2022;17(4):16–32. 10.17650/1818-8346-2022-17-4-16-32. (In Russ.).

[49] Tsai JW, Tsou JH, Hung LY, Wu HB, Chang KC. Combined Erdheim-Chester disease and Langerhans cell histiocytosis of skin are both monoclonal: a rare case with human androgen-receptor gene analysis. J Am Acad Dermatol. 2010;63(2):284–91. 10.1016/j.jaad.2009.08.013. 20633799 10.1016/j.jaad.2009.08.013

[50] Auerbach A, Aguilera NS. The changing landscape of pediatric histiocytoses: Birth, life, and transdifferentiation of pediatric histiocyte. Semin Diagn Pathol. 2023;40(6):420–8. 10.1053/j.semdp.2023.05.003. 37258365 10.1053/j.semdp.2023.05.003

[51] Pegoraro F, Kondyli M, Prudowsky ZD, van Halteren AGS. Histiocyte Society blueprint for Langerhans cell histiocytosis research: from cell of origin to a more comprehensive cure. Haematologica. 2025;110(11):2588–602. 10.3324/haematol.2024.286478. 41178438 10.3324/haematol.2024.286478 PMC12580689

[52] Misery L, Rougier N, Crestani B, Faure M, Claudy A, Schmitt D, et al. Presence of circulating abnormal CD34 + progenitors in adult Langerhans cell histiocytosis. Clin Exp Immunol. 1999;117(1):177–82. 10.1046/j.1365-2249.1999.00950.x. 10403933 10.1046/j.1365-2249.1999.00950.x PMC1905468

[53] Tesi M, Pegoraro F, Peyronel F, Emile JF, Catamerò F, Koster MJ, et al. Cluster analysis reveals the clinical spectrum of Erdheim-Chester disease. Leukemia. 2025;39(8):1987–96. 10.1038/s41375-025-02656-w. 40437172 10.1038/s41375-025-02656-w PMC12865793

[54] Raghav PK, Gangenahalli G. Hematopoietic Stem Cell Molecular Targets and Factors Essential for Hematopoiesis. J Stem Cell Res Ther. 2018;8(441):2. 10.4172/2157-7633.1000441.

[55] Rix B, Maduro AH, Bridge KS, Grey W. Markers for human haematopoietic stem cells: The disconnect between an identification marker and its function. Front Physiol. 2022;13:1009160. 10.3389/fphys.2022.1009160. 36246104 10.3389/fphys.2022.1009160 PMC9564379

[56] Chistiakov DA, Killingsworth MC, Myasoedova VA, Orekhov AN, Bobryshev YV. CD68/macrosialin: not just a histochemical marker. Lab Invest. 2017;97(1):4–13. 10.1038/labinvest.2016.116. 27869795 10.1038/labinvest.2016.116

[57] Duan M, Steinfort DP, Smallwood D, Hew M, Chen W, Ernst M, et al. CD11b immunophenotyping identifies inflammatory profiles in the mouse and human lungs. Mucosal Immunol. 2016;9(2):550–63. 10.1038/mi.2015.84. 26422753 10.1038/mi.2015.84 PMC7101582

[58] Schmid MC, Khan SQ, Kaneda MM, Pathria P, Shepard R, Louis TL, et al. Integrin CD11b activation drives anti-tumor innate immunity. Nat Commun. 2018;9(1):5379. 10.1038/s41467-018-07387-4. 30568188 10.1038/s41467-018-07387-4 PMC6300665

[59] Emile JF, Fraitag S, Leborgne M, de Prost Y, Brousse N. Langerhans’ cell histiocytosis cells are activated Langerhans’ cells. J Pathol. 1994;174(2):71–6. 10.1002/path.1711740202. 7965409 10.1002/path.1711740202

[60] de Graaf JH, Tamminga RY, Kamps WA, Timens W. Expression of cellular adhesion molecules in Langerhans cell histiocytosis and normal Langerhans cells. Am J Pathol. 1995;147(4):1161–71. 7573361 PMC1871002

[61] Carrera Silva EA, Nowak W, Tessone L, Olexen CM, Ortiz Wilczyñski JM, Estecho IG, et al. CD207+CD1a+ cells circulate in pediatric patients with active Langerhans cell histiocytosis. Blood. 2017;130(17):1898–902. 10.1182/blood-2017-05-782730. 28847997 10.1182/blood-2017-05-782730

[62] Kumar M, Updesh Singh Sachdeva M, Naseem S, Ahluwalia J, Das R, Varma N, et al. Bone marrow infiltration in Langerhan’s cell histiocytosis - An unusual but important determinant for staging and treatment. Int J Hematol Oncol Stem Cell Res. 2015;9(4):193–7. 26865930 PMC4748689

[63] Grandchamp B, Hetet G, Kannengiesser C, Oudin C, Beaumont C, Rodrigues-Ferreira S, et al. A novel type of congenital hypochromic anemia associated with a nonsense mutation in the STEAP3/TSAP6 gene. Blood. 2011;118(25):6660–6. 10.1182/blood-2011-01-329011. 22031863 10.1182/blood-2011-01-329011

[64] Liu D, Yi S, Zhang X, Fang P, Zheng C, Lin L, et al. Human STEAP3 mutations with no phenotypic red cell changes. Blood. 2016;127(8):1067–71. 10.1182/blood-2015-09-670174. 26675350 10.1182/blood-2015-09-670174 PMC4768430

[65] Wang X, Zhou Y, Ning L, Chen J, Chen H, Li X. Knockdown of ANXA10 induces ferroptosis by inhibiting autophagy-mediated TFRC degradation in colorectal cancer [published correction appears. Cell Death Dis. 2023;14(10):683. 10.1038/s41419-023-06151-x. 10.1038/s41419-023-06151-x PMC10579223 37845204

[66] Li Y, Liu Y, Wu P, Tian Y, Liu B, Wang J, et al. Inhibition of Ferroptosis Alleviates Early Brain Injury After Subarachnoid Hemorrhage In Vitro and In Vivo via Reduction of Lipid Peroxidation. Cell Mol Neurobiol. 2021;41(2):263–78. 10.1007/s10571-020-00850-1. 32314126 10.1007/s10571-020-00850-1 PMC11448630

[67] Fang Y, Gao S, Wang X, Cao Y, Lu J, Chen S, et al. Programmed Cell Deaths and Potential Crosstalk With Blood-Brain Barrier Dysfunction After Hemorrhagic Stroke. Front Cell Neurosci. 2020;14:68. 10.3389/fncel.2020.00068. 32317935 10.3389/fncel.2020.00068 PMC7146617

[68] Montalbetti N, Simonin A, Kovacs G, Hediger MA. Mammalian iron transporters: families SLC11 and SLC40. Mol Aspects Med. 2013;34(2–3):270–87. 10.1016/j.mam.2013.01.002. 23506870 10.1016/j.mam.2013.01.002

[69] Mayr R, Janecke AR, Schranz M, Griffiths WJ, Vogel W, Pietrangelo A, et al. Ferroportin disease: a systematic meta-analysis of clinical and molecular findings [published correction appears in J Hepatol. 2011;55(3):734-6. 10.1016/j.jhep.2011. 10.1016/j.jhep.2010.05.016 PMC2956830 20691492

[70] Szklarczyk D, Kirsch R, Koutrouli M, Nastou K, Mehryary F, Hachilif R, et al. The STRING database in 2023: protein-protein association networks and functional enrichment analyses for any sequenced genome of interest. Nucleic Acids Res. 2023;51(D1):D638–46. 10.1093/nar/gkac1000. 36370105 10.1093/nar/gkac1000 PMC9825434

